# MAD2L2 dimerization and TRIP13 control shieldin activity in DNA repair

**DOI:** 10.1038/s41467-021-25724-y

**Published:** 2021-09-14

**Authors:** Inge de Krijger, Bastian Föhr, Santiago Hernández Pérez, Estelle Vincendeau, Judit Serrat, Alexander Marc Thouin, Vivek Susvirkar, Chloé Lescale, Inés Paniagua, Liesbeth Hoekman, Simranjeet Kaur, Maarten Altelaar, Ludovic Deriano, Alex C. Faesen, Jacqueline J. L. Jacobs

**Affiliations:** 1 Division of Oncogenomics, The Netherlands Cancer Institute, Amsterdam, The Netherlands; 2 Laboratory of Signal Dynamics, Max-Planck Institute for Biophysical Chemistry, Göttingen, Germany; 3 Genome Integrity, Immunity and Cancer Unit, Equipe Labellisée Ligue Contre Le Cancer, INSERM U1223, Institut Pasteur, Paris, France; 4 Université de Paris, Sorbonne Paris Cité, Paris, France; 5 Proteomics Facility, The Netherlands Cancer Institute, Amsterdam, The Netherlands; 6 Biomolecular Mass Spectrometry and Proteomics, Utrecht Institute for Pharmaceutical Sciences, University of Utrecht, Utrecht, The Netherlands

**Keywords:** Chromosomes, DNA damage and repair

## Abstract

MAD2L2 (REV7) plays an important role in DNA double-strand break repair. As a member of the shieldin complex, consisting of MAD2L2, SHLD1, SHLD2 and SHLD3, it controls DNA repair pathway choice by counteracting DNA end-resection. Here we investigated the requirements for shieldin complex assembly and activity. Besides a dimerization-surface, HORMA-domain protein MAD2L2 has the extraordinary ability to wrap its C-terminus around SHLD3, likely creating a very stable complex. We show that appropriate function of MAD2L2 within shieldin requires its dimerization, mediated by SHLD2 and accelerating MAD2L2-SHLD3 interaction. Dimerization-defective MAD2L2 impairs shieldin assembly and fails to promote NHEJ. Moreover, MAD2L2 dimerization, along with the presence of SHLD3, allows shieldin to interact with the TRIP13 ATPase, known to drive topological switches in HORMA-domain proteins. We find that appropriate levels of TRIP13 are important for proper shieldin (dis)assembly and activity in DNA repair. Together our data provide important insights in the dependencies for shieldin activity.

## Abstract (web summary)

MAD2L2 — a member of the shieldin complex — is known to play important roles in DNA repair. Here the authors demonstrate how MAD2L2 dimerization mediated through SHLD2 participates in shieldin assembly and function.

## Introduction

MAD2L2, for MAD2-Like2, interacts with a variety of proteins to act in multiple important cellular processes. Initially identified as REV7, MAD2L2 directly interacts with REV3 and, together with two accessory proteins POLD2 and POLD3, forms Polymerase ζ (Polζ)^1^. Polζ functions in DNA damage tolerance by working with REV1 in translesion synthesis (TLS)-mediated bypass of replication blocking lesions^1^. In addition, MAD2L2 interacts with the anaphase-promoting complex/cyclosome (APC/C) activator CDH1 to prevent premature mitotic exit, and localizes to mitotic spindles and chromosomes through interactions with CHAMP1, RAN GTPase and others^2–4^. Moreover, MAD2L2 acts at DSBs and uncapped telomeres, where it forms a complex called “shieldin”, with the recently characterized SHLD1, SHLD2, and SHLD3 factors^5–13^. Shieldin functions in DNA repair by localizing to the exposed DNA ends and counteracting 5’ end-resection^5–14^. By doing so, shieldin complex members, including MAD2L2, promote DNA repair through non-homologous end-joining (NHEJ) while inhibiting DNA repair through homologous recombination (HR)^15^.

The mode of action that MAD2L2 employs in these different protein complexes is remarkably similar. MAD2L2 is a relatively small protein and consists mainly of a HORMA domain, originally defined by sequence conservation among the yeast proteins Hop1, Rev7, and Mad2^16^. HORMA binding to an interacting motif in its client protein promotes a striking topological remodeling of the HORMA domain from an “open” to a “closed” conformation of its “safety-belt”, a structurally mobile element comprising the last ~50 residues of the protein. This process is best understood for spindle assembly checkpoint (SAC) protein MAD2, where the considerable activation energy to be invested in the structural changes results in the creation of the very stable, albeit temporary, signaling complex. The structural conversion of MAD2 is catalyzed, both at the assembly and the disassembly level, by specialized protein machinery, allowing dynamic control of signaling. As part of that machinery, AAA + -ATPase TRIP13 has been shown to convert “closed” MAD2 to “open” MAD2^17–20^. Core properties of MAD2 signaling are conserved in MAD2L2, including the capture of peptides in a closed conformation and dimerization. Indeed, MAD2L2 was found to entrap peptides of different interactors, including REV3, CHAMP1 and RAN GTP, through its safety-belt^3,21,22^. These proteins interact with MAD2L2 through a REV7 Binding Motif (RBM), a consensus sequence defined as ϕϕxPxxxpPSR^21,23,24^. REV3 contains two of these RBMs, that both are important for the function of Polζ in ICL-repair^24^. Interestingly, MAD2L2 is able to bind to both RBMs on REV3 simultaneously and forms a REV3-mediated homodimer, which is important for TLS^25,26^.

Within the shieldin complex, MAD2L2 directly interacts with SHLD3^12,27^. This interaction relies on an RBM motif in SHLD3 and is abolished by mutations in the MAD2L2 safety-belt region^12,28^. Hence it resembles the interaction between MAD2L2 and REV3. Interestingly, and again in analogy to the REV3-interaction, SHLD3 contains two RBM motifs^12^. However, different from REV3, only one of the SHLD3 RBMs (RBM2) was found important for shieldin function, raising uncertainty on whether MAD2L2 dimerizes in shieldin. We therefore set out to study the importance of MAD2L2 dimerization in the assembly and function of shieldin. Interestingly, we find that abolishing the ability of MAD2L2 to dimerize prevents MAD2L2 from interacting with SHLD2 and impairs its function in NHEJ. Indeed, a recent report showing the crystal structure of a MAD2L2-SHLD3^1-64^-SHLD2^1-52^ complex revealed a MAD2L2 dimer within shieldin^29^. Interestingly, analogous to MAD2, the AAA + family ATPase TRIP13 was recently shown to facilitate the opening of the MAD2L2 safety-belt, thereby affecting shieldin complex formation^30^. Indeed, we find that TRIP13 interacts with MAD2L2 in the shieldin complex. We show that this critically depends on SHLD2-driven MAD2L2 dimerization and SHLD3, and that TRIP13 affects shieldin (dis)assembly and DNA repair. Altogether, our work reveals that appropriate activity of shieldin depends on MAD2L2 dimerization to drive appropriate assembly and disassembly of shieldin, facilitated by regulatory activity by TRIP13.

## Results

### MAD2L2 forms a dimer mediated by SHLD2 and important for shieldin assembly

**Fig. 1:**
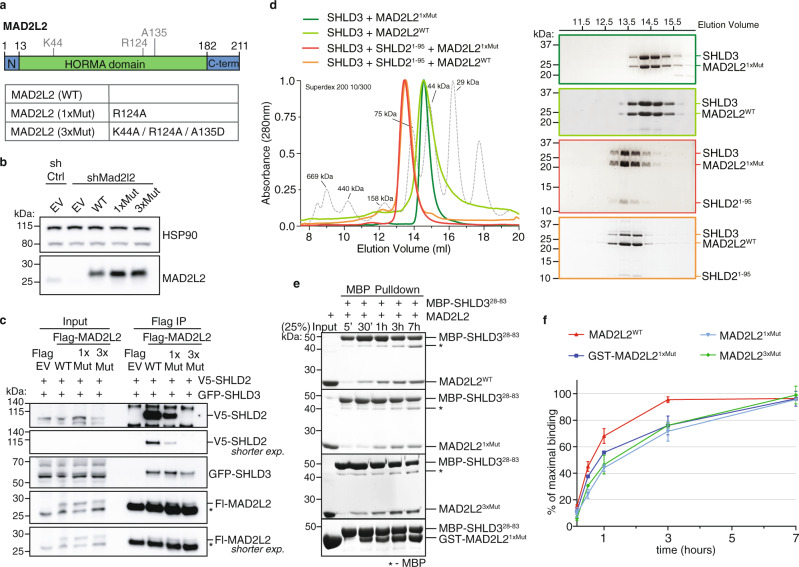
MAD2L2 forms a dimer mediated by SHLD2 that is important for shieldin assembly. **a** Schematic representation of the MAD2L2 protein with residues mutated to abolish dimerization. **b** Immunoblot of TRF2ts MEFs transduced with indicated shRNAs, complemented with shRNA-resistant expression constructs (EV, empty vector control; WT, full-length wild-type MAD2L2; 1xMut and 3xMut as indicated in **a**, all with single Flag-tag). HSP90 serves as loading control. **c** Immunoblot of 293T cells transfected with indicated epitope-tagged constructs followed by Flag immunoprecipitation. V5-SHLD2 is detected with V5-antibody, GFP-SHLD3 with GFP-antibody and Flag-MAD2L2 (Fl-MAD2L2) with MAD2L2-antibody. Asterisk denotes immunoglobulin light-chain. Representative of three independent experiments. **d** SHLD2^1-95^ induces dimerization of MAD2L2-SHLD3 complex. Coomassie-stained SDS-PAGE gels of SEC-profiles of SHLD3-MAD2L2 complex (WT: light green, 1xMut: dark green) and SHLD3-SHLD2^1-95^-MAD2L2 complex (WT: red, 1xMut: orange). Shift in elution-profile indicates an apparent increase of molecular weight of ~54 to ~90 kDa upon addition of SHLD2^1-95^. Molecular weight of MAD2L2 is 25 kDa, of SHLD3 is 29 kDa and of SHLD2^1-95^ is 11 kDa. **e** Time course of shieldin assembly between components MAD2L2 and SHLD3 shows that assembly of SHLD3-MAD2L2 complex is slow, but accelerated by MAD2L2 dimerization. Coomassie-stained SDS-PAGE gels show a time course of pulldowns of MBP-SHLD3 (bait) and MAD2L2-variants (prey). The SHLD3-fragment used here contains only the second RBM, which is captured by the seatbelt of MAD2L2^29^. A single-point mutation at R124 (1xMut) hinders MAD2L2 dimerization and subsequently lowers binding kinetics. Triple mutation of residues K44, R124, and A135 (3xMut) in the dimerization interface of MAD2L2 has the same effect as 1xMut on binding kinetics. Inducing dimerization by creating GST-fusion partially rescues the assembly kinetics. Representative of *n* = 4 (MAD2L2^WT^/MAD2L2^1*x*M*ut*^) or *n* = 2 (MAD2L2^3*x*M*ut*^/GST-MAD2L2^1*x*M*ut*^). **f** Graphical representation of data in **e**. Red curve represents binding kinetics between MAD2L2^WT^ and SHLD3^28-83^ peptide. Light blue and green curves represent the reduction in binding kinetics of MAD2L2^1*x*M*ut*^ and MAD2L2^3*x*M*ut*^ with SHLD3^28-83^. Dark blue curve represents partial restoration of binding kinetics between MAD2L2^1*x*M*ut*^ and SHLD3^28-83^ induced by dimerization of the GST-tag. Graphs represent mean ± s.e.m, *n* = 4 for MAD2L2^WT^/MAD2L2^1*x*M*ut*^, *n* = 2 for MAD2L2^3*x*M*ut*^/GST-MAD2L2^1*x*M*ut*^. Source data are provided as a Source Data file.

To understand to what extent MAD2L2’s ability to dimerize affects its function in shieldin we introduced dimer-breaking mutations in a flag-tagged RNAi-resistant (RR) MAD2L2 cDNA expression construct. We introduced an R124A amino acid substitution, originally used to disrupt homodimerization of MAD2L2 in crystallography studies and known to maintain MAD2L2 safety-belt interactions^3,12,21,27,31^. In addition, we changed two additional residues, K44A and A135D, that were recently identified to localize to a defined MAD2L2 dimerization-surface^25^. We thereby obtained expression constructs encoding for MAD2L2 harboring a single amino acid substitution (MAD2L2^R124A^; 1xMut) or three (MAD2L2^K44A/R124A/A135D^; 3xMut) (Fig. 1a and Supplementary Fig. 1a). We complemented MAD2L2*-*depleted cells with the different shRNA-resistant wild-type or mutant MAD2L2 expression constructs and confirmed their expression. As expected based on previous reports^25,31^, the mutations did not impair MAD2L2 protein stability (Fig. 1b). To address whether MAD2L2 dimerization affects shieldin complex formation we focused on the two shieldin proteins that directly interact with MAD2L2: SHLD3 and SHLD2. SHLD3 interacts with MAD2L2 through the MAD2L2 safety-belt, and SHLD2 interacts with MAD2L2-SHLD3 through its N-terminal region^9,10,12,27^. SHLD1, on the other hand, participates in shieldin by interacting with the C-terminal domain of SHLD2^10,27^. Thus, any impact on the interaction between MAD2L2 and SHLD2 would also affect the participation of SHLD1 in shieldin. We co-transfected 293T cells with epitope-tagged SHLD2, SHLD3, and wild-type MAD2L2 or the different MAD2L2-mutants and immunoprecipitated MAD2L2 in order to assess its interaction with SHLD2 and SHLD3. Interestingly, a single R124A amino acid substitution in MAD2L2 (1xMut) strongly reduced its ability to interact with SHLD2, but in line with previous in vitro data on MAD2L2^R124A^-SHLD3 interaction^27,28^, did not disturb the interaction of MAD2L2 with SHLD3 (Fig. 1c). Moreover, three single amino acid changes in the dimerization domain of MAD2L2 completely disrupted its ability to interact with SHLD2, while only slightly affecting its interaction with SHLD3 (Fig. 1c). This indicates that in vivo MAD2L2 dimerization is necessary for efficient interaction of MAD2L2 with SHLD2, but is largely dispensable for MAD2L2-SHLD3 interaction. Indeed, size-exclusion chromatography (SEC) on purified shieldin proteins indicated that SHLD2^1-95^ induces the dimerization of MAD2L2 in vitro, suggesting that a dimer of MAD2L2 is present in a full-length shieldin complex interacting with single molecules of SHLD3 and SHLD2 (Fig. 1d and Supplementary Fig. 1b, c). Of note, both the wild-type and MAD2L2 (1xMut) appear as monomers in the SEC experiment shown in Fig. 1d. This is expected as the reported dimerization affinity of MAD2L2 is weak^25^, and proteins were applied in low concentrations.

In MAD2, dimerization is known to accelerate the slow, but spontaneous structural conversion from the open to closed topology required for MAD2 incorporation into the Mitotic Checkpoint Complex MCC^32^. In vitro, MAD2 conversion and MCC assembly requires several hours^33–36^. Indeed, when mixing MAD2L2 with SHLD3 we observed similar slow complex assembly. The kinetics of complex assembly were further attenuated when using a dimerization deficient MAD2L2 (1xMut, or 3xMut) (Fig. 1e, quantified in f). This could subsequently be partially rescued by forcing dimerization of MAD2L2 (1xMut) through a GST fusion (Fig. 1e, f, see Supplementary Fig. 1d for the GST-MAD2L2 dimer). Altogether, these results indicate that within shieldin, MAD2L2 interacts with SHLD3 as a dimer, and that MAD2L2 dimerization is induced by binding of SHLD2. This is in line with recent structural data showing MAD2L2 as a dimer in the shieldin complex^29^.

### Efficient NHEJ at telomeres and in CSR requires MAD2L2 dimerization

**Fig. 2:**
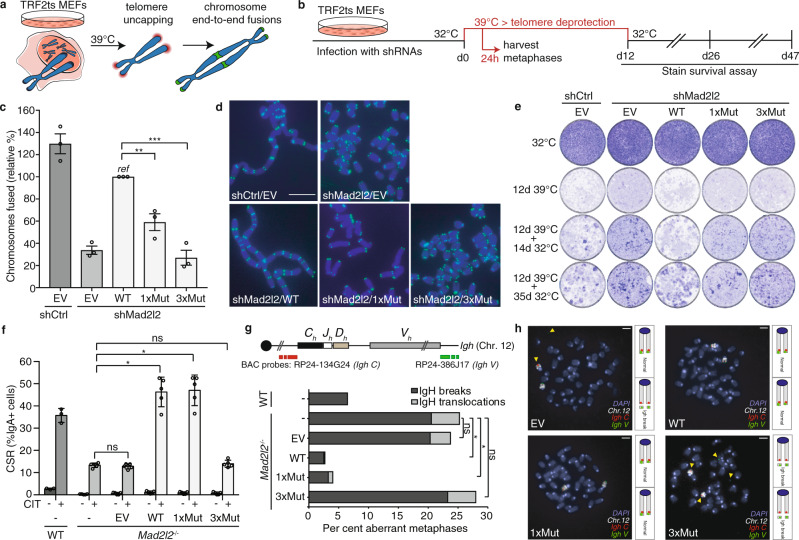
MAD2L2 dimerization is needed for MAD2L2-mediated control of NHEJ at deprotected telomeres and MAD2L2-mediated class switch recombination. **a** Experimental approach to address telomere deprotection induced NHEJ. **b** Schematic overview of the experimental setup used in **c**–**e. c** Chromosomal fusions in TRF2ts MEFs transduced with indicated shRNAs and expression constructs (*n* = 3 independent experiments, mean ± s.e.m., a minimum of 1500 chromosomes were counted per condition, per experiment). One-way ANOVA with Dunnett’s multiple comparisons test (**p = 0.0046; ***p = 0.0002). **d** Representative images of metaphases counted in **c**. Scale bar, 10 μm. **e** Survival assay of TRF2ts MEFs used in **c**–**d** Cells were cultured at 32 °C to exclude toxicity of the infected cells, or at 39 °C for 12 days to induce telomere deprotection. Cells were returned to 32 °C for the indicated times, and stained to assess survival. Plates are representative of *n* = 2 independent biological duplicates, the second biological duplicate is shown in Supplementary Fig. 1e, f. **f** CSR defect in *Mad2l2*^−/−^ cells is rescued after complementation with WT MAD2L2 and 1xMut but not with 3xMut. Bars represent mean ± s.e.m., two-sided Wilcoxon–Mann –Whitney test. *n* = 3 independent experiments with one WT clone and two *Mad2l2*^−/−^ clones, ns *p* = 0.6752 (-vs. EV) and *p* = 0.8340 (-vs. 3xMut); *p = 0.0119. CIT: anti-CD40, IL-4 and TGF-β. **g** Quantification of *Igh* breaks and translocations in metaphases of the indicated WT and *Mad2l2*^−/−^ CH12F3 cells. Horizontal bars represent means, two-sided Fisher’s exact test; *n* = 200 metaphases (WT), *n* = 400 (*Mad2l2*^−/−^), *n* = 384 (*Mad2l2*^−/−^ + EV), *n* = 400 (*Mad2l2*^−/−^ + WT), *n* = 400 (*Mad2l2*^−/−^ + 1xMut), *n* = 357 (*Mad2l2*^−/−^ + 3xMut) from one experiment (WT) or two independent experiments (*Mad2l2*^−/−^), ns *p* = 0.9798 (-vs. EV) and *p* = 0.9721 (-vs. 3xMut); *p = 0.0146 (-vs. WT) and *p* = 0.0126 (-vs. 1xMut). **h** Representative images of *Igh* breaks in aberrant metaphases in *Mad2l2*^−/−^ CH12F3 cells, as quantified in **g**. Scale bars, 5 μm. Significance; ns, not significant (*p* ≥ 0.05); *p < 0.05, **p < 0.01, ***p < 0.001. Source data are provided as a Source Data file.

To address the functional importance of MAD2L2 dimerization in DNA repair, we tested the ability of the different MAD2L2 dimerization-mutants to promote NHEJ. For this we made use of *Trf2*^−/*−*^*;p53*^−/*−*^ mouse embryonic fibroblasts (MEFs) harboring a temperature-sensitive allele of the telomeric shelterin component TRF2 (TRF2ts) that provide a system for assessing ligase 4-dependent classical NHEJ at natural chromosome ends following TRF2-inactivation mediated telomere deprotection^37^. While these TRF2ts MEFs grow fine at the permissive temperature of 32 °C, culturing them at non-permissive temperatures (37–39 °C) causes TRF2 to dissociate from telomeres, leaving telomeric chromosome ends deprotected and causing them to elicit a DNA damage response. This results in processing of the deprotected telomeres through NHEJ, giving rise to chromosome end-to-end fusions, and eventually cell death due to genomic crisis^38^ (Fig. 2a). Depletion of factors critical for NHEJ, including MAD2L2 and other shieldin components, prevents chromosomal fusions and cell death^5,9,27,38^. To assess the impact of disrupting MAD2L2 dimerization on NHEJ, we restored expression of MAD2L2 in MAD2L2-depleted TRF2ts MEFs with wild-type or dimerization mutant MAD2L2 cDNAs and quantified chromosome end-to-end fusions upon 24 h of telomere uncapping at the non-permissive temperature (Fig. 2b). As demonstrated before^5^, chromosomal fusions upon telomere deprotection in MAD2L2-depleted cells were strongly diminished, indicative of defective NHEJ (Fig. 2c, d). Complementation of MAD2L2-depleted cells with exogenous full-length wild-type MAD2L2 restored chromosome end-to-end fusion (Fig. 2c, d). Interestingly however, the single MAD2L2^R124A^ mutant (1xMut), that partially lost SHLD2 interaction, was significantly impaired in its ability to support chromosomal fusion upon telomere deprotection. Moreover, in line with the complete loss of MAD2L2-SHLD2 interaction, the triple MAD2L2^K44A/R124A/A135D^ mutant (3xMut) was completely defective in promoting chromosome end fusion in a background of endogenous MAD2L2 depletion (Fig. 2c, d), indicating that this dimerization mutant cannot support NHEJ at telomeres. These findings were recapitulated in an assay for cell survival upon prolonged telomere uncapping (Fig. 2b, e and Supplementary Fig. 1e, f). In line with our previous work^5^, control cells expressing endogenous MAD2L2 died of crisis upon prolonged telomere uncapping, while MAD2L2 depletion allowed these cells to survive. Consistent with their ability to promote telomere fusion, this survival was abolished by re-introducing full-length wild-type MAD2L2, but not by the single and triple MAD2L2-mutants (Fig. 2e and Supplementary Fig. 1e, f).

In addition, we addressed the ability of the different MAD2L2-mutants to promote NHEJ during immunoglobulin class switch recombination (CSR), a physiological process that relies on classical NHEJ-mediated joining of activation-induced cytidine deaminase-mediated DSBs (Supplementary Fig. 2a for experimental setup). As we demonstrated before^5,9^, MAD2L2-deficient cells are strongly impaired in CSR. This CSR defect could be fully restored by complementing MAD2L2-deficient cells with exogenous wild-type MAD2L2 (WT) or with MAD2L2^R124A^ (1xMut), but not with MAD2L2^K44A/R124A/A135D^ (3xMut) that lost the capability to interact with SHLD2 in the shieldin complex (Fig. 2f and Supplementary Fig. 2b–d). These findings were further recapitulated by assessing the ability of MAD2L2 to prevent *Igh* breaks and translocations in CH12F3 cells. While *Igh* breaks and translocations were strongly increased in MAD2L2-deficient CH12F3 cells, these were restored to normal low levels by complementation with wild-type MAD2L2 (WT) or MAD2L2^R124A^ (1xMut), but not with MAD2L2^K44A/R124A/A135D^ (3xMut), indicating that these residues are critical for proper MAD2L2 function at DNA breaks at the *Igh* locus (Fig. 2g, h). Interestingly, our finding that NHEJ during CSR, but not telomere-NHEJ, can still be fully supported by the dimerization-defective MAD2L2 (1xMut), indicates that CSR and telomere-NHEJ have different requirements. These might relate to differences in the underlying biology. We hypothesize that for CSR, in which AID induces a DSB at a defined locus, the low level of shieldin assembly that still takes place with the 1xMut is sufficient to maintain functional AID-DSB repair. CSR might therefore be less sensitive to partial defective shieldin complex, such as with the MAD2L2 (1xMut), compared to telomere-NHEJ in which multiple telomeres get deprotected at the same time. Moreover, as 30-50% of CSR can still occur independently of ligase-4/XRCC4-dependent NHEJ, it is possible that in a situation with partially defective shieldin complex some of the breaks are repaired through alternative end-joining.

Altogether this indicates that the interactions mediated by the K44, R124 and A135 residues on MAD2L2, involved in MAD2L2 dimerization, are critical for the ability of MAD2L2 to function in NHEJ.

### The ATP-ase TRIP13 interacts with shieldin in a MAD2L2 dimerization and SHLD3-dependent manner

**Fig. 3:**
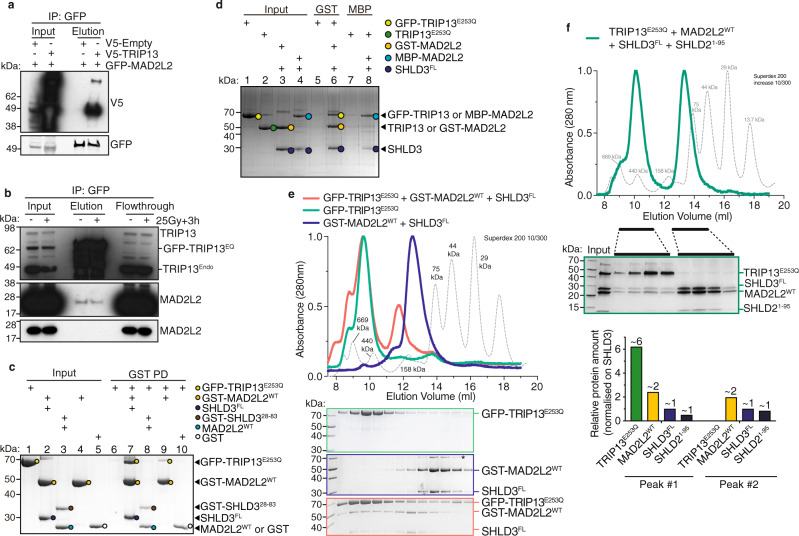
TRIP13 interacts with dimerized MAD2L2 in shieldin. **a** Immunoblot of GFP-IP in 293T cells transfected with the indicated constructs. Representative of two independent experiments. **b** Immunoblot of GFP-IP in unirradiated or irradiated (25 Gy+3 h) HeLa cells expressing dox-inducible GFP-tagged TRIP13-EQ. Representative of two independent experiments. **c** GST-tagged MAD2L2 in complex with full-length SHLD3 (lane 7) interacts directly with TRIP13 while the absence of SHLD3 strongly reduces the interaction with TRIP13 (lane 9). Representative of five independent experiments. **d** Interaction with TRIP13 is dependent on MAD2L2 dimerization (induced here by a GST-tag, which is known to dimerize^61^). GST-tagged MAD2L2 in complex with SHLD3 (lane 6), but not MBP-tagged MAD2L2 in complex with SHLD3 (lane 8), interacts directly with TRIP13. Representative of five independent experiments. **e** TRIP13-MAD2L2-SHLD3 form a stable complex. Coomassie-stained SDS-PAGE gels of SEC profiles of TRIP13 (green), MAD2L2-SHLD3 (blue), and TRIP13-MAD2L2-SHLD3 (red). When mixed, the elution-profiles of TRIP13 and MAD2L2-SHLD3 shift to an apparent higher molecular weight, indicative of complex formation. MAD2L2-SHLD3 can interact to both the monomer TRIP13 and the hexamer TRIP13. All experiments were performed in presence of 0.1 mM ATP. Asterisk denotes contaminant. Molecular weights of GST-MAD2L2 is 52 kDa, of SHLD3 is 29 kDa and of GFP-TRIP13 is 75.5 kDa (453 kDa for hexamer). Representative of three experiments. **f** MAD2L2-SHLD3-SHLD2^1-95^ interacts stably with a TRIP13^E253Q^ hexamer. Coomassie-stained SDS-PAGE gels of SEC profile of TRIP13-MAD2L2-SHLD3-SHLD2^1-95^. Proteins were mixed in equimolar concentrations. All experiments were performed in the presence of 0.1 mM ATP to induce hexamerisation of TRIP13^E253Q^. Molecular weights are: MAD2L2 = 24 kDa, SHLD3 = 29 kDa, SHLD2^1-95^ = 10.3 kDa, and TRIP13^E253Q^ = 48.5 kDa (291 kDa for hexamer). Representative of five experiments. Source data are provided as a Source Data file.

To obtain more insight in shieldin complex assembly we set out to identify MAD2L2 interacting proteins with a potential role in MAD2L2 complex formation. For this, we immunoprecipitated MAD2L2 from HeLa cells depleted for endogenous MAD2L2 and stably expressing GFP-tagged MAD2L2, and analyzed co-precipitating peptides by mass spectrometry. Cells were either left untreated or harvested 3 h after inducing DNA damage by irradiation (IR) with 25 Gy. Aside from SHLD2 and SHLD3, we identified multiple additional interacting proteins (Supplementary Fig. 3a). Among these, the AAA + family ATPase TRIP13 in particular caught our attention. One reason for this being its known interaction with MAD2 and other HORMA-domain containing proteins^39^. The other reason being that TRIP13 was among positive hits in the same functional genetic screen for regulators of telomere-induced genomic instability, that originated our identification of MAD2L2 as regulator of NHEJ, suggesting that TRIP13 acts in similar processes as MAD2L2 (Supplementary Fig. 3b, c)^5^. Indeed, we could confirm an interaction between MAD2L2 and TRIP13 in co-immunoprecipitation assays with exogenous epitope-tagged MAD2L2 and TRIP13, as well as with endogenous MAD2L2 in cells expressing GFP-tagged ATPase-dead TRIP13^EQ^ (Fig. 3a, b). Interestingly, MAD2L2 dimerization appears critical for interaction with TRIP13. In vitro, MAD2L2 directly interacts with TRIP13, but only when dimerization was induced by a GST-tag on MAD2L2 or by the presence of SHLD2^1-95^ (Fig. 3c, d, f, also see Supplementary Fig. 1d). In addition, interaction of TRIP13 with MAD2L2 requires full-length SHLD3 to be part of the complex. In absence of full-length SHLD3 the interaction of a MAD2L2 dimer with TRIP13 was only very weak or non-detectable (Fig. 3c, compare lanes 7 and 9). Furthermore, SEC revealed that, analogous to MAD2, a hexamer of ATPase activity deficient TRIP13^E253Q^ forms a stable complex with MAD2L2-SHLD3 and MAD2L2-SHLD3-SHLD2^1-95^ in the presence of ATP in vitro (Fig. 3e, f). Interestingly, while TRIP13 association with MAD2 requires p31^comet^ as a cofactor^39^, the formation of a stable TRIP13-shieldin complex apparently does not need a cofactor. However, p31^comet^ does seem important for the activity of TRIP13 towards shieldin^40^.

Altogether, our in vivo and in vitro analysis indicate that TRIP13 interacts with shieldin in a manner requiring MAD2L2 dimerization supported by SHLD2, and SHLD3.

### TRIP13 affects DNA repair

We next addressed the functional relevance of the interaction between TRIP13 and shieldin. We hypothesized that similar to what is known for MAD2^17–20^, TRIP13 might facilitate a conformational switch in MAD2L2, providing the energy to open up MAD2L2 and release its interacting partner(s). This would allow MAD2L2 to shuttle between different protein complexes and activities, or facilitate release of shieldin activity after DNA repair has advanced or completed. Moreover, by promoting shieldin-disassembly, TRIP13 could promote HR and inhibit NHEJ at the right place and time. Hence, disturbing such a role by TRIP13 might impact DNA repair. To address this we overexpressed exogenous wild-type (WT) or catalytic inactive (ATPase-dead) TRIP13 (TRIP13^EQ^), or depleted endogenous TRIP13 in different cell lines. We confirmed effective ablation of TRIP13 and functionality of exogenous wild-type or EQ-mutant TRIP13 expression by making use of the notion that a functional SAC requires TRIP13 ATPase-activity^41^ (Supplementary Fig. 4a–e). Indeed, unlike TRIP13 proficient cells, cells completely deficient for TRIP13 failed to arrest in mitosis upon mitotic spindle disruption with nocodazole, indicating a dysfunctional SAC. Exogenous expression of wild-type TRIP13, but not TRIP13^EQ^, restored the ability of TRIP13 knockout cells to arrest in mitosis.

**Fig. 4:**
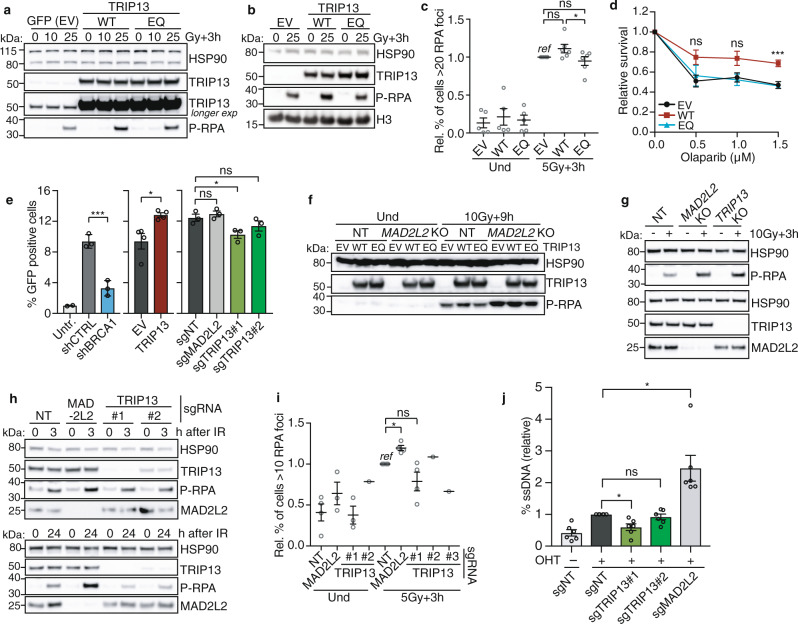
TRIP13 affects DNA repair. **a** Immunoblot of HeLa cells transduced with wild-type TRIP13 (WT), ATPase-dead TRIP13 (EQ) or GFP-control vector (EV), 3 h post IR (10/25 Gy). HSP90 as loading control. Representative of *n* = 3. **b** Immunoblot of U2OS cells transduced as in **a** (25 Gy+3 h), representative of *n* = 4. **c** RPA-foci in U2OS transduced as indicated, untreated (Und) or 3 h post IR (5 Gy), normalized to control (EV). Dots represent *n* = 6 biological replicates, except “Und” (*n* = 5), 115–600 cells analyzed/condition/replicate, mean ± s.e.m., one-way ANOVA, Tukey’s multiple comparisons test on IR-treated samples (ns *p* = 0.2109 (EV vs. WT) and *p* = 0.6638 (EV vs. EQ); *p = 0.0385). **d** Survival of U2OS transduced as **b**, **c**, treated with olaparib for 19 days (*n* = 4 biological replicates, showing mean ± s.e.m., one-way ANOVA, Dunnett’s multiple comparisons test for each concentration, significance shown for EV vs. WT, ns *p* = 0.1166 (0.5μM) and *p* = 0.0846 (1.0μM); ***p = 0.0007 (1.5μM)). **e** DR-GFP (HR) assay in U2OS, shRNAs/sgRNA infected or transfected with untagged-TRIP13 (EV, empty vector control). DSBs were induced with I-SceI, except for “Untr”. Dots show independent replicates, mean ± s.e.m., one-way ANOVA (Dunnett’s) (shRNA/sgRNA-treated cells, *n* = 3 except “Untr” (*n* = 2), ***p = 0.0006; ns *p* = 0.0720 (sgNT vs. sgMAD2L2) and *p* = 0.7091 (sgNT vs. sgTRIP13#2); *p = 0.0332) or unpaired two-tailed *t*-test (TRIP13 overexpression set, *n* = 4, *p = 0.0173). **f** Immunoblot of transduced control (NT, non-target) or *MAD2L2* KO U2OS cells, untreated (Und) or 9 h post 10 Gy. **g** Immunoblot of long-term polyclonal *TRIP13* KO U2OS cells, a *MAD2L2* KO U2OS clone and NT control cells, harvested 3 h post IR. Representative of *n* = 5. **h** Immunoblot of transduced U2OS cells, untreated (0 h) or 3 h and 24 h post IR (10 Gy). Representative of *n* = 3. **i** RPA-foci in U2OS transduced as in **h**, untreated (Und) or 3 h + 5 Gy, normalized to sgNT. Dots represent *n* = 4 individual experiments, except sgTRIP13#2/3 (*n* = 1), 100–600 cells/condition/replicate, mean ± s.e.m., one-way ANOVA (Dunnett’s) for sgNT, sgMAD2L2, sgTRIP13#1 (ns *p* = 0.2516; *p = 0.0140). **j** Quantification of single-stranded DNA in RT–PCR-based end-resection assays in sgRNA-transduced U2OS-DIvA cells after DSB induction with 4-hydroxytamoxifen (OHT) or untreated (-). Mean ± s.e.m., *n* = 6, one-way ANOVA (Dunnett’s) (ns *p* = 0.7939; *p = 0.0332 (sgNT vs. sgTRIP13#1) and *p* = 0.0445 (sgNT vs. sgMAD2L2). Significance; ns, not significant (*p* ≥ 0.05), *p < 0.05, ***p < 0.001. Source data are provided as a Source Data file.

We first investigated if TRIP13 affects DNA end-resection, as MAD2L2 and shieldin factors are known to promote NHEJ and inhibit HR by counteracting DNA end-resection. Interestingly, stable overexpression of wild-type TRIP13, in both Hela and U2OS cells, resulted in increased IR-induced phosphorylation of the single-stranded DNA binding protein RPA on Ser4/8 (p-RPA), indicative of elevated end-resection (Fig. 4a, b). On the other hand, overexpression of ATPase-dead TRIP13^EQ^ did not, or to much lesser extent, increase p-RPA. As TRIP13 functions as a hexamer, we hypothesize that the overexpression of TRIP13^EQ^ in the presence of endogenous TRIP13 can contribute to the formation of TRIP13-hexamers with partial activity. In addition, IR-induced foci formation by RPA appeared slightly elevated (not reaching statistical significance) upon overexpression of wild-type TRIP13, but not TRIP13^EQ^, in U2OS cells (Fig. 4c). As TRIP13 enhanced DNA end-resection in our assays, we hypothesized that overexpression of TRIP13, by facilitating end-resection, would render cells more efficient in HR. To address this, we treated U2OS cells expressing WT or EQ-mutant TRIP13 with the PARP-inhibitor olaparib and addressed survival, as resistance to PARP inhibition is dependent on HR. Indeed, overexpression of WT TRIP13, but not the EQ mutant, caused enhanced resistance to treatment with olaparib (Fig. 4d and Supplementary Fig. 5a). In line with this, cells overexpressing TRIP13 showed enhanced HR efficiency in a DR-GFP reporter assay (Fig. 4e and Supplementary Fig. 5b). Finally, we addressed whether the ability of TRIP13 to enhance end-resection depends on MAD2L2. As expected from earlier work^5,6^, depletion of MAD2L2 resulted in elevated IR-induced p-RPA at 3 h post IR (Fig. 4f). While overexpression of TRIP13 enhanced p-RPA in MAD2L2-proficient cells, it did not further enhance end-resection in a MAD2L2 KO background, indicating that TRIP13 controls end-resection in a MAD2L2-dependent manner. Thus, opposite of the activity of MAD2L2 that counteracts resection and HR, TRIP13 promoted DNA end-resection and HR when overexpressed. This is in line with the interaction we observed between MAD2L2 and TRIP13, with a regulatory activity of TRIP13 on MAD2L2 that is related to the topological switches it induces in HORMA proteins and with a recent report showing that TRIP13 overexpression promotes shieldin-disassembly^30^.

As we reasoned that also absence of TRIP13 might interfere with proper MAD2L2 function we addressed the consequences of CRISPR/Cas9-mediated TRIP13-deficiency for DNA end-resection in stable polyclonal *TRIP13* KO U2OS cells, in clonal *TRIP13* KO Hela and U2OS cell lines, and upon short-term polyclonal depletion of TRIP13 with lentiviral sgRNAs (Fig. 4g–j, Supplementary Figs. 4f and 5c, d, f, g). Interestingly, upon different conditions of long-term TRIP13 inactivation in U2OS and HeLa cells, IR-induced p-RPA levels were increased (Fig. 4g and Supplementary Fig. 5c). IR-induced RPA foci were also increased in both *MAD2L2* and *TRIP13* KO lines (Supplementary Figs. 4f and 5d). Of note, shRNA-mediated depletion of MAD2L2 could still further enhance p-RPA levels in a *TRIP13* KO background (Supplementary Fig. 5e). While this could indicate that shieldin-independent roles of TRIP13 contribute to the increased p-RPA in TRIP13-KO lines, it could also reflect more efficient interference with shieldin function that is potentially not complete with either shRNA-mediated depletion of MAD2L2 (despite being very efficient) or TRIP13 KO alone. Conversely, and in line with previous reported findings^30^, efficient but not full disruption of TRIP13 with independent lentiviral sgRNAs was generally associated with signs of reduced end-resection, showing a slight decrease in p-RPA levels and RPA foci in MEFs and U2OS at 3 or 24 hrs post IR (Fig. 4h, i and Supplementary Fig. 5f, g). As RPA responses were mild and for RPA foci not reaching statistical significance over limited number of replicates, we further addressed the effect of TRIP13 depletion on resection by using a PCR-based resection assay^42,43^. As expected, the amount of ssDNA was enhanced in absence of MAD2L2. TRIP13 depletion had the opposite effect and decreased the amount of resection (Fig. 4j and Supplementary Fig. 5h). In line with this, TRIP13 depletion also decreased HR-efficiency in a DR-GFP reporter assay (Fig. 4e). So while long-term complete TRIP13 disruption appears associated with signs of elevated end-resection (Fig. 4g and Supplementary Figs. 4f and 5c–e), short-term TRIP13 depletion seems associated with decreased resection (Fig. 4h–j and Supplementary Fig. 5f, g). We hypothesize that the observed increase in RPA or p-RPA in long-term TRIP13 KO cells reflects disturbed shieldin complex dynamics, or might be a consequence of shieldin-independent roles of TRIP13, as further eluted to in the discussion below.

Overall, the effects of TRIP13 on resection were less prominent than of MAD2L2, which could well be a reflection of manipulation of a factor that affects the kinetics of shieldin (dis)assembly being less severe than removing a component of shieldin itself. Also, this would be consistent with MAD2L2 spontaneously but slowly disassembling from its interactors over time without the need for TRIP13, in line with the known spontaneous but very slow disassembly of MAD2L2-SHLD3 and other HORMA interactions^28,34^.

Given the disturbed end-resection control in TRIP13-depleted cells, we assessed sensitivity to olaparib, both in wild-type cells and in BRCA1-deficient cells that are hypersensitive to olaparib due to HR-deficiency. Efficient depletion of TRIP13 in U2OS or RPE-WT cells did not clearly affect the sensitivity to olaparib (Supplementary Fig. 6a, b). However, both shRNA- and sgRNA-mediated depletion of TRIP13 in BRCA1-deficient RPE cells rendered these cells less sensitive to olaparib (Supplementary Fig. 6c, d). Although less pronounced, this effect of TRIP13 depletion resembled that of MAD2L2 depletion in BRCA1-deficient cells, observed in the same assays and as reported before^6^. As PARP inhibitor resistance upon depletion of MAD2L2 in BRCA1-deficient cells is known to be mediated by restoration of end-resection and HR^6^, this could indicate that HR is also partially restored in TRIP13-depleted BRCA1-deficient cells. This is in contrast to a recent report showing enhanced sensitivity to PARP inhibitors in TRIP13-depleted BRCA1-deficient SUM149PT cells^30^ and to the decreased HR and resection in BRCA1-proficient U2OS cells we observed upon TRIP13 depletion. These differences might be due to different cell types used, as cells differ in the amount of free MAD2L2 to engage in different protein complexes, further eluted to in the discussion. They may also relate to the signs of elevated resection we observe with long-term depletion of TRIP13 that might come through in these survival assays of longer duration and might result from impaired shieldin complex dynamics or shieldin-independent effects. Furthermore, as MAD2L2 has a prominent role in ICL-repair, we addressed the sensitivity of *TRIP13* KO cells to cisplatin induced ICLs. Interestingly, *TRIP13* KO U2OS cells displayed modest sensitivity to cisplatin (Supplementary Fig. 7a). Although this did not reach statistical significance over 3 replicates, it may indicate that TRIP13 to some extent also impacts other MAD2L2-functions, such as in ICL-repair.

**Fig. 5:**
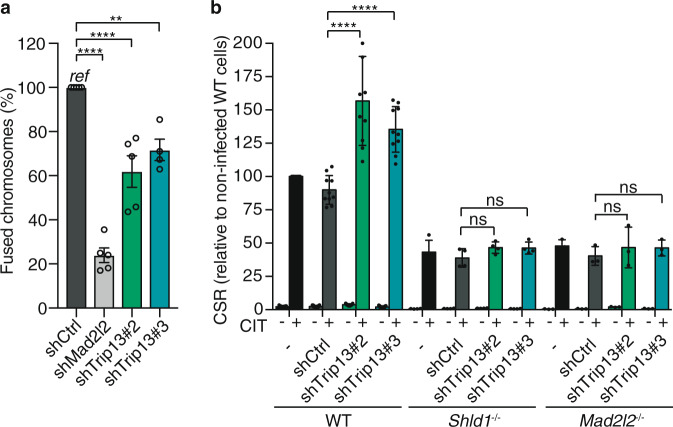
TRIP13 affects NHEJ-mediated DNA repair. **a** Chromosome fusions in TRF2ts MEFs infected with indicated shRNAs, after 24 h of telomere uncapping. Values are relative to control infected cells (shCtrl). Dots represent individual experiments (*n* = 5, except shTrip13#3 (*n* = 4), mean ± s.e.m., >1500 chromosomes counted per condition per experiment), one-way ANOVA, Dunnett’s multiple comparisons test (**p = 0.0022; ****p < 0.0001). **b** CSR is increased in absence of TRIP13, using two different shRNAs, but not in a *Shld1-* and *Mad2l2*-null background. Mean ± s.e.m. relative to non-infected WT cells, two-sided Wilcoxon–Mann–Whitney test. *n* = 10 independent experiments except *Shld1*^−/−^ (*n* = 4), and *Mad2l2*^−/−^ (*n* = 3). ****p < 0.0001; ns *p* = 0.1143 (shCtrl vs. shTrip13#2) and *p* = 0.3429 (shCtrl vs. shTrip13#3) for *Shld1*^*−/−*^*;* ns *p* = 1 (shCtrl vs. shTrip13#2) and *p* = 0.4 (shCtrl vs. shTrip13#3) for *Mad2l2*^*−/−*^. Significance; ns, not significant (*p* ≥ 0.05), **p < 0.01, ****p < 0.0001. Source data are provided as a Source Data file.

Finally, we studied the role of TRIP13 at the level of NHEJ. Overexpression of TRIP13 in TRF2ts MEFs did not significantly affect the amount of chromosome end-to-end fusions upon telomere uncapping (Supplementary Fig. 7b, c), suggesting that it did not interfere with the ability of MAD2L2 to promote telomere-NHEJ. However, depletion of TRIP13 using two different shRNAs significantly reduced the amount of chromosomal fusions, albeit to lesser extent than the depletion of MAD2L2 did (Fig. 5a and Supplementary Fig. 7d). This is in line with TRIP13 being among the hits of the same shRNA screen that originally identified MAD2L2 as positive regulator of NHEJ and relies on cell survival upon prolonged telomere uncapping as proxy for impaired NHEJ at telomeres, as extensive fusion of uncapped telomeres causes p53-deficient cells to die of genomic crisis^5^ (Supplementary Fig. 3b, c). Interestingly, opposite from the effect on telomere-NHEJ, depletion of TRIP13 raised NHEJ during CSR (Fig. 5b and Supplementary Fig. 7e–h). Moreover, the increased CSR-efficiency was dependent on SHLD1 and MAD2L2 (Fig. 5b), indicating that TRIP13 depletion increases CSR-efficiency through the shieldin complex. Thus, while this clearly indicates that in absence of TRIP13, normal control of NHEJ is perturbed, NHEJ at telomeres and at immunoglobulin loci are affected differently. We hypothesize that this might be due to differences in the biology underlying telomere-NHEJ and CSR, or might be related to additional roles for TRIP13 in the context of telomere-NHEJ, as we will further discuss below.

Together our data indicate that aberrant TRIP13 levels, both too much or too little, interfere with normal control of DNA repair, with the outcome for DNA repair being context dependent.

### TRIP13 controls the assembly of SHLD2/3 at DNA breaks

**Fig. 6:**
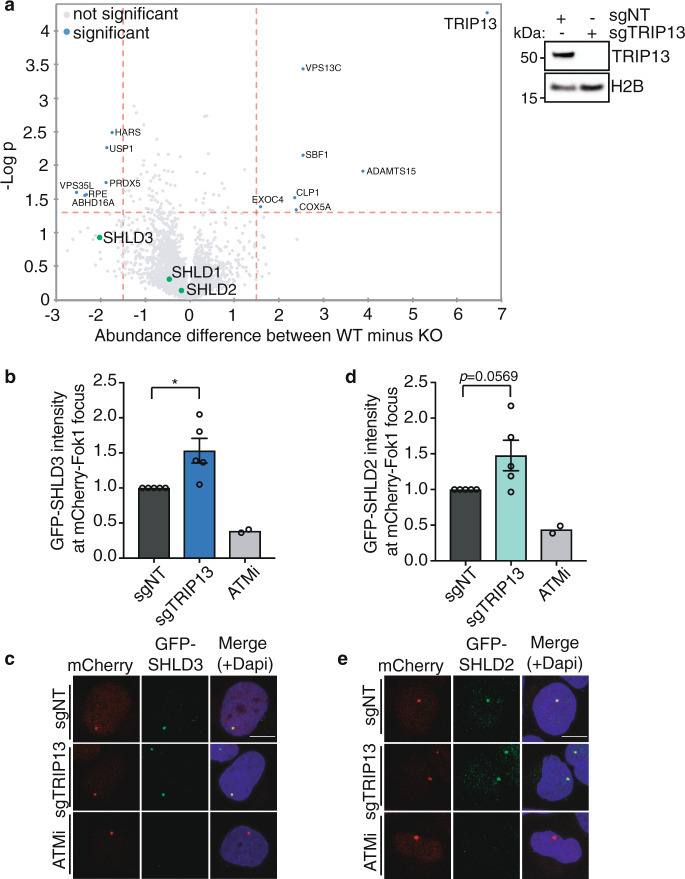
TRIP13 controls the assembly of SHLD2/3 at DNA breaks. **a** Left: Mass-Spectrometry analysis of MAD2L2 interactions upon pulldown of 3xflag-tagged MAD2L2 in 293T cells transduced with sgNT or sgTRIP13. Vulcanoplot represents the abundance difference between the sgNT (WT) and sgTRIP13 (KO) cells and significance over *n* = 3 independent pulldown experiments. Right: Immunoblot of 293T cells used for mass-spectrometry analysis. **b** Quantification of the intensity of GFP-tagged SHLD3 signal at mCherry-LakI-Fok1 foci 3 h post activation of the Fok1 nuclease by addition of 4-OHT and shield-1. Graph represent the mean ± s.e.m. of *n* = 5 (except for ATMi-treated conditions for which *n* = 2) relative to the sgNT transduced cells. A minimum of 129 cells were analyzed per condition per experiment. Significance was calculated using an unpaired two-tailed *t*-test, *p = 0.0168. **c** Representative image of cells quantified in **b**. **d** Quantification of the intensity of GFP-tagged SHLD2 as in **b**. A minimum of 88 cells were analyzed per condition per experiment (two-tailed unpaired *t*-test, ns *p* = 0.0569). **e** Representative image of cells quantified in **d**. Scale bar, 10 μm. Significance; ns, not significant (*p* ≥ 0.05), *p < 0.05. Source data are provided as a Source Data file.

As DNA repair related processes under control by shieldin were affected by TRIP13, we hypothesized that aberrant expression of TRIP13 impairs shieldin complex dynamics. To address this we studied shieldin complex formation in the absence of TRIP13. We expressed 3xflag-tagged MAD2L2 in 293T cells depleted for TRIP13 or in control cells (sgNT), followed by MAD2L2-immunoprecipitation and mass-spectrometry analysis of immunoprecipitated complexes (Fig. 6a). Interestingly, while the interaction of MAD2L2 with SHLD1 and SHLD2 was not affected, the interaction of MAD2L2 with SHLD3 seemed enhanced in TRIP13 KO cells, despite the difference not being statistically significant over 3 replicate experiments (Fig. 6a). Since SHLD3 is interacting with MAD2L2 through the MAD2L2 safety-belt, the SHLD3-MAD2L2 interaction is the most likely interaction in shieldin to be affected by TRIP13, that would be expected to be stabilized in the absence of TRIP13. To address this we studied the recruitment of the different shieldin complex members to sites of broken DNA in the presence or absence of TRIP13. For this we expressed GFP-tagged SHLD2 or SHLD3 in U2OS-DSB reporter cells, in which DNA breaks are induced with 4-OHT and shield-1 inducible mCherry-LacI-Fok1 nuclease^44^, and depleted TRIP13 (Fig. 6b–e and Supplementary Fig. 8a–c). As expected, as shieldin localization to DSBs depends on ATM kinase activity^5,7^, ATM-inhibitor treatment strongly decreased GFP-SHLD2 and GFP-SHLD3 foci intensity at mCherry-marked DSBs. Interestingly, cells depleted for TRIP13 displayed increased GFP-SHLD3 and GFP-SHLD2 accumulation at DSBs. This illustrates that in absence of TRIP13 accumulation of shieldin at DNA DSBs is increased and indicates that TRIP13 affects the recruitment or the stability of the shieldin complex at DNA breaks.

## Discussion

MAD2L2 engages in multiple complexes to coordinate essential cellular processes, including TLS by participating in Polζ and DSB repair by being a central component of shieldin. By doing so MAD2L2 affects the development and treatment of human disease, such as Fanconi Anemia and cancer, in which it determines the sensitivity of BRCA1-deficient cancers to treatment with Parp inhibitors. Given these critical roles, understanding the requirements for proper MAD2L2 activity is important. Here, we show how dimerization of MAD2L2 is critical for shieldin complex formation and activity in DNA repair and is promoted by SHLD2. Moreover, MAD2L2 dimerization and the presence of SHLD3 allow the shieldin complex to interact with the ATPase TRIP13, which is important for the appropriate dynamics of shieldin complex assembly and appropriate control of DNA repair reactions.

For the function of MAD2L2 in TLS the formation of a REV3-tethered MAD2L2-homodimer, facilitated by two RBM motifs in REV3, is essential^24,25^. SHLD3 also contains two putative RBM motifs, however only one was found important for the function of shieldin^12,28^. Whether the formation of a functional shieldin complex would involve and require dimerization of MAD2L2 was therefore not immediately obvious. Here we show that MAD2L2 indeed forms a dimer within the shieldin complex. This dimer formation is important for the assembly of shieldin and is stabilized by the SHLD2 component. We find that in absence of MAD2L2 dimerization, SHLD2 cannot participate in the shieldin complex in vivo. This finding explains a previous reported result on a K129A-mutation in MAD2L2 that reduced the interaction of SHLD3-MAD2L2 to SHLD1 and SHLD2^12^, as this mutation is located in the MAD2L2 dimerization domain. In line with its necessity for productive shieldin assembly, we show that MAD2L2 dimerization is essential for the function of MAD2L2 in the repair of deprotected telomeres through NHEJ, as well as the appropriate NHEJ-mediated joining of DSBs in immunoglobulin loci during CSR. While completing this manuscript, the dimerization of MAD2L2 was also revealed in the crystal structure of MAD2L2-SHLD3^1-64^-SHLD2^1-52^, showing that a dimer of MAD2L2 molecules is embraced by a peptide of SHLD3^29^. At the C-terminus of the SHLD3^1-64^ peptide, the RBM forms the canonical safety-belt with the MAD2L2 molecule, while the N-terminus of the peptide forms a novel safety-belt interaction with the other MAD2L2 molecule that is mediated by the SHLD2 peptide. This clarifies why previously only one of the SHLD3 RBMs was found important for shieldin function, while we find here that dimerization of MAD2L2 is critical for its in vivo function in shieldin. In addition, our finding that SHLD2 supports the dimerization of MAD2L2 in vitro, is in line with the proposal from the structural data that SHLD2 primes the non-canonical interaction between the SHLD3 N-terminus and MAD2L2.

Importantly, we show that MAD2L2 dimerization not only promotes interactions within shieldin but also brings MAD2L2/shieldin the opportunity to interact with the TRIP13 ATPase. TRIP13 was recently reported to induce the dissociation of MAD2L2-SHLD3 through opening the MAD2L2 safety-belt and subsequent release of SHLD3^30^, indicating that in analogy to MAD2, TRIP13 regulates MAD2L2 complex disassembly in the context of shieldin. Our initial biochemical reconstitution efforts highlighted surprising differences in the molecular mechanism of TRIP13 on shieldin, when compared to the MCC. In the MCC, p31^comet^, itself in a permanent closed conformation, is an essential cofactor for MCC binding by bridging MAD2 to TRIP13^18,45^. This is in contrast to shieldin, where we observed no necessity for p31^comet^ in binding to TRIP13. p31^comet^, however, does seem important in mediating TRIP13-activity towards the shieldin complex^40^. It seems likely that the closed MAD2L2, that interacts with RBM2 in SHLD3 via the canonical safety-belt interaction, will be the equivalent of the closed MAD2. Interestingly, the second MAD2L2, of which the seatbelt is invisible in the recent structure^29^, but the core fold suggests that it too is in a closed conformation, might therefore take over the role of (closed) p31^comet^ in bridging TRIP13 towards the shieldin complex. Additionally, we observed that SHLD3 contributes to the interaction to TRIP13 outside of RBM2. It is currently unclear if SHLD3 provides direct binding to TRIP13, or whether longer segments are needed to fully stabilize the dimerization and thus interaction with TRIP13. Future detailed reconstitution efforts and structural studies will be necessary to explain the functional relevance of these differences.

Indeed, we find that the assembly of shieldin at DSBs and DNA repair reactions are sensitive to the presence and activity of TRIP13. Overexpression of TRIP13^WT^ but not the ATP-ase dead TRIP13^EQ^ increased the amount of ssDNA, increased HR-efficiency, and rendered cells resistant to PARP-inhibition. Moreover, depletion of TRIP13 stabilized SHLD3-MAD2L2 interaction, increased SHLD2 and SHLD3 accumulation at DSBs, decreased HR-efficiency and ssDNA formation, and enhanced NHEJ during CSR. This is in line with recent work^30^ and supports that in analogy to the activity of TRIP13 towards other HORMA interactions, enhanced TRIP13 activity dissociates shieldin, thereby dismantling its inhibitory activity to end-resection, while lack of TRIP13 activity stabilizes shieldin. However, we also observed seemingly opposite effects of TRIP13 depletion on DNA repair, such as signs of elevated end-resection in long-term TRIP13 KO cells, olaparib resistance in BRCA1-/-RPE cells and, in line with an earlier notion of impaired NHEJ in TRIP13-deficient cells^46^, impaired telomere-NHEJ. These observations indicate that the consequences of TRIP13 depletion are less straightforward and might reflect a more widespread effect of TRIP13 depletion on MAD2L2 interactions that may enhance shieldin function but may also negatively affect shieldin function, depending on context and read-out. We hypothesize that this is a reflection of MAD2L2 being a bi-stable system in which TRIP13 deficiency can yield two results that differentially impact reactions involving MAD2L2, depending on context and specific requirements of those reactions. On one hand the absence of TRIP13 slows down disassembly of shieldin and might therefore improve certain DNA repair reactions as it gives a more constitutive shieldin complex. However, in absence of TRIP13 promoting disassembly of MAD2L2 from the different complexes it engages in, MAD2L2 would also be locked in its interaction with partner proteins, such as shieldin, Polζ, CHAMP1 and others. We envision that this would strongly impair the ability of MAD2L2 to shuttle between the different complexes it participates in, reduce or ablate the free pool of MAD2L2 available to engage in specific complexes and activities when needed, and interfere with the temporal nature of MAD2L2 complex activity that potentially underlies normal control of the biological processes it operates in. Hence, by interfering with normal release and temporary action of MAD2L2 containing complexes, TRIP13 deficiency could both enhance and impair MAD2L2 controlled processes. The outcome might also be cell type-dependent as cells differ in amounts of free MAD2L2 and the distribution of MAD2L2 over different complexes. It might also be process dependent as different processes have different requirements due to the underlying biology. For instance, telomere end-joining relies heavily on ligase 4/XRCC4 and on the joining of distant deprotected chromosome ends that need to find each other, while end-joining in CSR can still occur for 30-50% in absence of ligase 4/XRCC4 and involves cis-joining events of switch-junctions brought together within CSR centers^47–49^. Affecting the MAD2L2-SHLD1/2/3 complex dynamics through modulation of TRIP13 levels might have different outcomes based on these differences. Cell cycle dependencies might also factor in the different outcomes; as NHEJ is preferred in G1, it is possible that the activity of TRIP13 is mostly important in S/G2 to promote efficient HR by inducing shieldin complex disassembly. In addition, it is also possible that some of the effects observed with TRIP13 deficiency are a consequence of other roles of TRIP13, outside of regulatory activity towards MAD2L2.

**Fig. 7:**
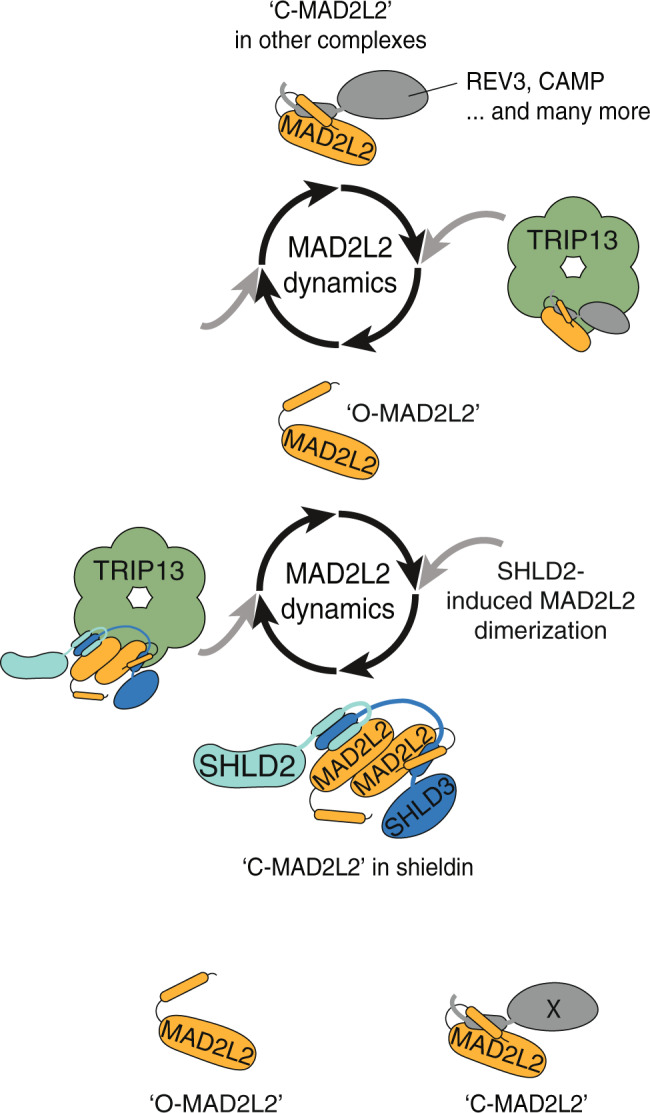
Model of MAD2L2 dynamics. Model of how MAD2L2 dynamics is regulated by SHLD2-induced MAD2L2 dimerization and TRIP13-mediated opening of the MAD2L2 safety-belt and release of the entrapped peptide. MAD2L2 can interact with various proteins by entrapping a specific peptide of its interacting partner with its C-terminal safety-belt, forming closed MAD2L2 (“C-MAD2L2”). TRIP13 facilitates the opening of the MAD2L2 safety-belt, promoting release of the MAD2L2 partner protein and resulting in open MAD2L2 (“O-MAD2L2”). TRIP13 might thereby contribute to the ability of MAD2L2 to shuttle between different protein complexes or promote disassembly of MAD2L2 protein complexes at the right place and time.

Overall, our data support a model in which dimerization of MAD2L2 is critical for shieldin assembly and allows TRIP13 to control appropriate dynamics and activity of the shieldin complex (Fig. 7). The modulation of the reversible topology switch in the MAD2L2 core structure by TRIP13 is expected to regulate its protein-protein interaction potential and kinetics. We therefore speculate that TRIP13 is part of a larger regulating network that will control that shieldin is assembled and disassembled in the right place and at the right time. TRIP13, as an enzymatic regulator of shieldin, has been proposed as an interesting candidate to explore as drug target to modulate Parp inhibitor responses^30^. However, the different outcomes we observed with TRIP13-deficiency and the regulation of multiple MAD2L2- or HORMA-complexes by TRIP13, indicate that this might not be straightforward and warrant thorough further exploration of effectiveness and potentially unwanted consequences.

## Methods

### Cell culture and survival assays

HeLa, U2OS, 293T, Phoenix (ATCC), DR-GFP U2OS^50^, AID-DIvA U2OS^51^, RPE, *BRCA1*^−/−^ RPE^52^ cells and *Trf2*^−/*−*^;*p53*^−/*−*^;TRF2(Ile468Ala) MEFs (TRF2ts MEFs)^37,38^ were cultured in Dulbecco’s modified Eagle medium with 10% FBS (Sigma), supplemented with 100 U penicillin, 100 μg ml^−1^ streptomycin and 2 mM l-Glutamine (Gibco, Life Technologies). CH12F3 (CH12)^53^ cell lines were cultured in RPMI 1640 supplemented with 10% FBS, 100 U ml^−1^ penicillin, 100 μg ml^−1^ streptomycin, 50 μM 2-mercaptoethanol, 1x MEM non-essential amino acids, 1 mM sodium pyruvate and 10 mM HEPES. HeLa, U2OS, RPE, 293Ts, Phoenix or CH12 cells were cultured at 37 °C, TRF2ts MEFs were cultured at the permissive temperature of 32 °C, all with 5% CO_2_. *BRCA1*^−/*−*^ RPE cells were maintained in a low oxygen (3%) incubator. To induce GFP-TRIP13 expression in HeLa cells, cells were treated with 0.5 μg ml^−1^ doxycycline for 24 h. To evaluate long-term survival upon prolonged telomere deprotection, TRF2ts MEFs were seeded at 40,000 cells/10 cm plate and from the next day cultured at the non-permissive temperature (39 °C) for 12 days. Cells were then returned to 32 °C for the indicated times, fixed with 4% formaldehyde and stained with 0.1% crystal violet. Crystal violet was extracted with 10% acetic acid and absorbance at 595 nm was measured in a Tecan microplate reader (Infinite M200pro, TECAN) for quantification.

### Virus production and constructs

For retrovirus production, phoenix producer cells were transfected with 20 μg plasmid DNA using standard calcium phosphate-DNA precipitation, and refreshed after 16 and 24 h. Supernatant containing viral particles was collected 48 and 62 h post transfection, filtered, supplemented with 4 μg ml^−1^ polybrene and added to the cells^38^. For lentivirus production, 293T cells were transfected with 10 μg plasmid DNA^5^. TRF2ts MEFs were transduced with pLKO-puro shRNA lentiviruses from Mission library clones (Sigma) targeting mouse *Mad2l2* (TRCN0000012846: 5′-CATCTTCCAGAAGCGCAAGAA-3′) or a scrambled control hairpin (5′-CAACAAGATGAAGAGCACCAA-3′). For complementation experiments, pMSCVblas retroviral vectors were used containing a shRNA-resistant wild-type MAD2L2 (MAD2L2^RR^) with a single flag-tag, described before^5^, or an empty vector control. The R124A, K44A and A135D mutations were generated using the QuikChange Site-Directed Mutagenesis Kit (Agilent). Primers are listed in Supplementary Table 1. For TRIP13 overexpression experiments, cells were transduced with lentiviruses containing pLX304-blast-V5-Empty, pLX304-blast-V5-GFP, pLX304-blast-V5-TRIP13, pMSCVblast or pMSCVblast-untagged hTRIP13. For the ATPase-mutant TRIP13 (TRIP13^EQ^), an E253Q mutation was generated in the pLX304-blast-V5-TRIP13 using the QuikChange Site-Directed Mutagenesis Kit (Agilent). For TRIP13 depletion, the following Mission Library clones (Sigma) pLKO-puro lentiviruses were used: mouse *Trip13* shRNA#2 (TRCN0000024990: 5′-CGTACTCTTCTCAGACAAGAA-3′); mouse *Trip13* shRNA#3 (TRCN0000024991: 5′-GAAGATATAAAGTCGAGTGTT-3′); human *TRIP13* shRNA#1 (TRCN0000022061: 5′-GCAGTGGACAAGCAGTTTGAA-3′), or human *TRIP13* shRNA#5 (TRCN0000022063: 5′-GCACTGTTGCACTTCACATTT-3′). For BRCA1 depletion, a pLKO-blast lentivirus was used targeting human *BRCA1*: TRC0000039837: 5′-GCCTACAAGAAAGTACGAGAT-3′. For CRISPR mediated knockout, the following sgRNA sequences were used and cloned into a pX330-puro or pLentiCRISPRv2 plasmid according to standard protocols: sgTRIP13#1: 5′-CGAGTCGCCAACGGTCCACG-3′(targeting exon 1), sgTRIP13#2: 5′-TGAGTAGCTTTCTAACACTC-3′ (targeting exon 2), sgTRIP13#3: 5′-CACGTGGACCGTTGGCGACT-3′(targeting exon 1), sgMAD2L2: 5′-GAGGTCTTGTCGTGTGAGCG-3′ (targeting exon 1), sgNT: 5′-GGTAGCGAACGTGTCCGGCG-3′ (for pLentiCRISPRv2) or sgNT: 5′-GGGTCTTCGAGAAGACCTGT-3′ (for pX330-puro). To obtain stable knockout clones, U2OS cells were seeded at 1 × 10^5^ cells/6-well plate and the next day transfected with the pX330-puro CRISPR gRNA constructs containing the individual guides using *Trans*IT-TL1 Transfection reagent (Mirus) and the next day selected with 4 μg ml^−1^ puromycin for 3–4 days. Next, cells were seeded in 96-well plates in puro-free medium and individual clones were picked and grown out. A polyclonal U2OS *TRIP13* KO cell line was made by pooling individual *TRIP13* KO clones.

PLentiCRISPRv2 was a gift from Feng Zhang (Addgene plasmid #52961)^54^. pX330-U6-Chimeric_BB-CBh-SpCas9 was a gift from Feng Zhang (Addgene plasmid #42230)^55^. Clones were made by transfecting a modified version of the pX330 backbone containing puromycin resistance^56^.

For experiments in FokI DSB reporter cells, SHLD2 and SHLD3 expression constructs were cloned into a pMSCVblast-eGFP retroviral backbone using Gateway technology (Invitrogen).

### Immunoblotting

Immunoblotting was done according to standard protocols and as described before^5^. Briefly, cells were washed with PBS and collected by scraping in 2xSDS sample buffer followed by boiling at 95 °C. Alternatively, cell extracts were made using an Urea-SDS buffer (6 M UREA, 1% SDS, 125 mM NaCl, 25 mM TRIS pH 8), collected with a cell scraper, sonicated for 15 s at 20% frequency and stored at −20 °C until further use. Protein concentrations were measured using Pierce BCA Protein Assay (Thermo Scientific) and equal amounts of protein were loaded onto precast 4–12% Bis-Tris gels (Invitrogen). The following antibodies were used: HSP90 (sc-7947, Santa-Cruz, 1:500), MAD2L2 (135977, Santa-Cruz, 1:500 and 12683-1-AP, ProteinTech, 1:500), GFP (A11122, Life Technologies, 1:1000), TRIP13 (ab128153, Abcam, 1:1000), V5 (R96025, Invitrogen, 1:500), H3 (ab1791, Abcam, 1:1000), H2B (07-371, Millipore, 1:1000), p-RPA (RPA2 pS4/pS8: NBP1-23017, Novus Biologicals, 1:500 and A300-245A, Bethyl, 1:1000), p-Kap1 (S824, A300-767A, Bethyl, 1:1000), γ-Tubulin (T6557, Sigma, 1:10,000). Membranes were incubated with horseradish peroxidase (HRP)-conjugated secondary antibodies (1:7500 goat anti-rabbit IgG HRP, G21234 or goat anti-mouse IgG HRP G21040, Invitrogen), for detection using enhanced chemiluminescence (SuperSignal West Pico PLUS, Thermo Scientific) on a Syngene G:BOX, or with IRDye800CW- and IRDye680-labeled secondary antibodies (1:10,000, IRDye800CW goat anti-mouse IgG 926-32210, IRDye800CW goat anti-rabbit IgG 926-32211, IRDye680 goat anti-mouse IgG 926-32220 or IRDye680 goat anti-rabbit IgG 926-32221, LI-COR) for detection on the Odyssey Infrared imager (LI-COR).

### Immunoprecipitations

For flag-immunoprecipitation experiments, 293T cells were seeded at 5 × 10^6^ cells/10 cm plate and transfected with 10 μg of the indicated combinations of tagged-cDNA constructs using standard CaPO_4_ transfection. Cells were collected 48 h post transfection and resuspended in 1 ml lysis buffer (50 mM Tris-HCl pH 7.4, 150 mM NaCl, 1 mM EDTA and 1% triton X-100) supplemented with protease- and phosphatase-inhibitors (cOmplete Tablets EDTA-free protease cocktail tablets, PhosStop, Roche), 10 mM iodoacetamide (I1149, Sigma) and 5 mM Sodium Butyrate (B5887, Sigma). Lysates were kept on ice for a total of 30 min while pipetting extensively every 10 min followed by centrifugation (20,000 × *g*) after which an input sample was taken. Lysates were added to anti-Flag M2 Magnetic beads (M8823, Sigma-Aldrich) pre-washed in TBS (50 mM Tris HCl, 150 nM NaCl pH 7.4) and incubated at 4 °C overnight. After washing with TBS, beads were resuspended in 2xSDS sample buffer and boiled at 95 °C for 5 min to elute immunocomplexes. For GFP-immunoprecipitation assays, cells expressing the indicated constructs were plated in 15 cm plates, irradiated or left untreated and collected in ice-cold PBS by scraping after washing three times with PBS. Immunoprecipitation was done using GFP-Trap beads (chromotek) essentially according to the manufacturers protocol. Cells were resuspended in ice-cold lysis buffer (10 mM TrisHCl pH 7.5, 150 mM NaCl, 0.5 mM EDTA, 0.5% NP-40), supplemented as above with protease- and phosphatase-inhibitors, iodoacetamide and Sodium Butyrate, and incubated on ice for 30 min while pipetting extensively every 10 min. Cell lysates were centrifuged (20,000 × g for 10 min), the pellet was discarded and the lysate was supplemented with dilution buffer (10 mM TrisHCl pH 7.5, 150 nM NaCl, 0.5 mM EDTA) supplemented with the inhibitors indicated above and an aliquot was taken as input sample. Beads were equilibrated by washing three times with dilution buffer, and added to the lysates followed by rotation overnight at 4 °C. After collecting the flow-through, beads were washed three times with dilution buffer. For elution of protein complexes, beads are resuspended in 100 μl 2xSDS-sample buffer and boiled for 10 min at 95 °C. For analysis by mass spectrometry, MAD2L2-depleted HeLa cells were used complemented with GFP-MAD2L2^RR^ or a GFP-control. One sub-confluent 15 cm plate (~1 × 10^7^ cells) was used per condition per experiment.

### Mass spectrometry for MAD2L2 interacting proteins

For mass spectrometry, samples were run short-distance on a 4–12% SDS-PAGE gel and stained with Coomassie Blue. The lane was excised from the gel after which proteins were reduced with dithiothreitol and alkylated with iodoacetamide. Proteins were digested with trypsin (mass spec grade, Promega) overnight at 37 °C and peptides were extracted with acetonitrile. Digests were dried in a vacuum centrifuge and reconstituted in 10% formic acid for MS analysis. Peptide mixtures (10% of total digest) were loaded directly on the analytical column and analyzed by nanoLC-MS/MS on an Orbitrap Fusion Tribrid mass spectrometer equipped with a Proxeon nLC1000 system (Thermo Scientific).

### Mass-spectrometry data analysis

Raw data were analyzed by Proteome Discoverer (PD) (version 2.3.0.523, Thermo Scientific) using standard settings. MS/MS data were searched against the Human Swissprot database (20,367 entries, release 2020_02) using Sequest HT. The maximum allowed precursor mass tolerance was 50 ppm and 0.6 Da for fragment ion masses. Trypsin was chosen as cleavage specificity allowing two missed cleavages. Carbamidomethylation (C) was set as a fixed modification, while oxidation (M) was used as variable modifications. False discovery rates for peptide and protein identification were set to 5% and as additional filter Sequest HT XCorr>1 was set. For the heatmap, samples were analyzed using the free R scripting language. The PD output file containing the PSM counts was loaded into R (version 4.0.2). Spectral counts of the biological replicates were averaged and proteins were filtered for zero counts in the control and for at least one count (average of two replicates) in one of the other two conditions. The counts were represented in a heat map with a color code ranging from white (0 PSM(s)) to red (≥5 PSM(s)). For the volcano plot, the PD output file containing the abundances was loaded into Perseus (version 1.6.14.0)^57^. LFQ intensities were Log2-transformed and the proteins were filtered for at least two out of three valid values. Missing values were replaced by imputation based on the standard settings of Perseus, i.e., a normal distribution using a width of 0.3 and a downshift of 1.8. Differentially expressed proteins were determined using a *t*-test (–LOG(*p*-value) ≥ 1.3 and [*x* – *y*] ≥ 1.5 | [*x* – *y*] ≤ −1.5).

### Immunofluorescence

For immunofluorescence, 40,000–60,000 U2OS cells/well were seeded in 8-well chamber slides (Millicell EZ slide, Millipore) and irradiated as indicated in the individual experiments. For RPA foci, cells were pre-extracted for 5 min by adding ice-cold 0.5% Triton/PBS on ice prior to fixing. Then, cells were washed with PBS, fixed for 10 min with 2% paraformaldehyde followed by 10 min permeabilization in 0.5% Triton/PBS. After 1 h of blocking (0.02% Triton, 5% NGS, 5% FCS in PBS), cells were incubated with primary antibody in blocking solution overnight at 4 °C. Primary antibodies used were RPA (RPA34-20, NA18, Calbiochem, 1:500). Next, cells were washed three times with 0.02% Triton/PBS and incubated with Alexa Fluor 488 or 568 goat anti-mouse or anti-rabbit IgG secondary antibodies (Invitrogen, 1:500) in blocking solution for 1 h. After washing three times with 0.02% Trition/PBS, slides were mounted using DAPI-containing Vectashield (Vector Laboratories).

For FokI DSB reporter experiments, U2OS ER-mCherry-LacI-FokI-DD cells^44^ were stably transduced with pMSCVbl-eGFP-SHLD2 or pMSCVbl-eGFP-SHLD3 prior to transduction with the indicated sgRNA constructs. Cells were treated with shield-1 (1 μM) and 4-OHT (0.5 μM) for 3 h and harvested as described above. In ATM inhibitor conditions, cells were treated 10 min prior to addition of shield-1 and 4-OHT with 10 μM of ATM Kinase inhibitor (KU55933, cat#118500, Calbiochem). Cells were stained with a primary antibody against GFP (A11122, Life Technologies, 1:500) and further processed as described above. Slides were imaged using a confocal Leica SP5 system with X63 oil objective and LAS-AF software (version 2.7.4). Image analysis was performed with a customized macro on ImageJ (version 1.52p) software and data was processed using Microsoft Excel (version 16.16.27).

### Metaphase chromosome analysis for telomere fusions

Cell harvesting, preparation of metaphases spreads and image acquisition was done as described before^5^. Briefly, *Trf2*^−/*−*^;*p53*^−/*−*^; TRF2ts MEFs were placed at the non-permissive temperature of 39 °C for 24 h to induce telomere deprotection. To enrich for mitotic cells, 10 μg ml^−1^ colcemid (Karyomax) was added to the cells 2 h prior to harvesting. Cells were collected by trypsinization, resuspended in 0.075 M KCl and incubated for 7 min at 37 °C followed by spinning down (5 min at 194 × *g*) and fixing in Methanol-Acidic Acid fixative. Telomere FISH was performed on chromatin spreads with an Alexa488-labeled (CCCTAA)3 (TelC) PNA probe (PN-TC060-005, Panagene/Eurogentec). Digital images of metaphases were captured using a Metafer4 (MetaSystems) equipped with an AxioImager Z2 microscope (Carl Zeiss)^5^.

### Flow cytometry

For pH 3 staining, cells were seeded at 1 × 10^6^ per 6 cm plate prior to adding 0.1 μg ml^−1^ nocodazole (Sigma) for 16 h. All cells were collected by trypsinization, washed with PBS and fixed by adding cold 100% EtOH. For staining, cells were washed with PBS-Tween (0,05% Tween) and anti-phosphoH3 Ser10 antibody (06-570, Millipore, 1:500) was added in PBS-Tween + 3% BSA for 2 h. After washing twice with PBS-Tween, cells were resuspended in PBST/3% BSA containing Alexa647 (Invitrogen, 1:500) for 1 h at room temperature. Finally, cells were washed twice with PBS-Tween, resuspended in PBS containing 250 μg ml^−1^ RNase and 10 μg ml^−1^ PI and incubated for 30 min at 37 °C. Samples were analyzed on a LSR II Flow Cytometer (BD Biosciences), gating on live and single cells (FSC/SSC) and analyzed using FlowJo (version 10).

### DR-GFP assay

Stable DR-GFP U2OS cells were a kind gift from Jeremy Stark^50^. To address HR-efficiency, 2 × 10^5^ cells were seeded in 12-well plates, 24 h prior to transfection with BFP and I-SceI using Lipofectamine 2000 (ThermoFisher). Cells were refreshed 3 h after transfection and harvested 72 h post transfection by trypsinization, followed by resuspension in PBS and flow cytometry analysis. Cells were gated for single cells and BFP, and the percentage of GFP-positive cells was quantified (Attune TM NxT Acoustic Focusing Cytometer, Thermo Fisher Scientific).

### PCR end-resection assay

The procedure was carried out as described previously^42,43^. Briefly, 1.5 × 10^6^ AID-AsiSI-ER-U2OS (AID-DIvA) cells^51^ were seeded in 10 cm plates 24 h before treatment with 0.3 µM 4-hydroxytamoxifen for 4 h. Genomic DNA was isolated using DNeasy Blood & Tissue kit with RNAse A addition (Qiagen), treated with RNAse H and digested or mock digested with BanI (New England Biolabs) overnight. BanI was then heat inactivated and 12.5 ng of the gDNA was used in a 10 μl quantitative PCR reaction (QuantStudio 6 Flex Real-Time PCR System, Applied Biosystems) in triplicate with SensiFAST™ SYBR® (Bioline) using primers flanking the BanI site 200 bp from the inducible AsiSI cut site (see Supplementary Table 1). The percentage of ssDNA was calculated as previously described^43^.

### Olaparib and cisplatin sensitivity assays

RPE or U2OS cells transduced with the indicated constructs were seeded at 1000–5000 cells/well (RPE or *BRCA1*^−/−^ RPE) or 800 cells/well (U2OS) into 6-well plates. For RPE, cells were seeded with either DMSO or the PARP inhibitor olaparib at the indicated concentrations. The medium was refreshed every 5 days, every condition in each experiment was seeded as a technical duplicate. For U2OS cells, olaparib was added at the indicated concentrations the day after seeding. For cisplatin sensitivity assays, 400 cells/well (U2OS) were seeded into 12-well plates as a technical duplicate for each experiment, and cisplatin was added at the indicated concentrations the day after seeding. After 10–12 days, cells were washed with PBS, fixed with 4% formaldehyde and stained using 0.1% crystal violet. The crystal violet was extracted using 10% acetic acid and quantified by measuring absorbance at 595 nm at a plate reader (Infinite M200pro, TECAN). For every condition, crystal violet intensity was normalized to the untreated (DMSO) control.

### CSR assay in CH12 cell lines

MAD2L2 WT, MAD2L2 R124A, and MAD2L2 3xMut(K44A/R124A/A135D) vectors were introduced by retroviral infection and CH12 cells were selected with blasticidin (10 μg ml^−1^). The aforementioned TRIP13-shRNA and control vectors were introduced by lentiviral infections and CH12 cells were selected with puromycin (3 μg ml^−1^). CH12 cells were subsequently plated at 50,000 cells/ml in complete RPMI supplemented with anti-CD40 antibody (1 μg ml^−1^, Miltenyi), IL-4 (20 ng ml^−1^, Miltenyi) and TGF-β (1 ng ml^−1^, R&D Biotech) to induce IgM to IgA switching. After 3–4 days, cells were assayed for class-switching by flow cytometry using an IgA-PE antibody (eBiosciences) and a Fortessa analyser (BD Biosciences). Viable cells were counted using a Casy cell counter (Roche). CSR and proliferation assays were done in at least three independent experiments.

### RT–PCR analysis

*Igh* α germ-line transcripts (αGLT) and *Aid* mRNA were quantified as previously described^9^. Briefly, total RNA was isolated from activated CH12 cells using the RNeasy mini kit (Qiagen) and cDNA was synthesized using the high capacity reverse transcription kit (Applied Biosystems). Semi-quantitative RT–PCR using 2.5-fold serial dilutions of cDNA was made from CH12 cells stimulated for 72 h with anti-CD40, IL-4, and TGF-β. *Hprt* was used as a control for transcript expression. Transcripts were amplified using oligonucleotides described in Supplementary Table 1.

### DNA FISH on metaphase spreads for *Igh* translocations

DNA FISH on metaphases spreads was performed as previously described^58^ using BAC probes RP24-134G24 (5’ Igh C) and RP24-386J17 (3’ Igh V) and XCyting Mouse Chromosome 12 (Orange) paint from MetaSystems. Metaphases were imaged using a ZEISS AxioImager.Z2 microscope and the Metafer automated capture system (MetaSystems), and counted manually.

### Expression of recombinant proteins and purification

All recombinant proteins used in biochemical reconstitution of complexes in this study were of human origin. MAD2L2^WT^, MAD2L2^1*x*M*ut*^, MAD2L2^3*x*M*ut*^ and SHLD3^28-83^ were expressed with an N-terminal GST/hexahistidine-MBP fusion-tag from pLIB at 16 °C in *E.coli* Rosetta strain for 16 h after induction with 0.5 mM IPTG. Cells were lysed by sonication in buffer A containing 25 mM HEPES (pH 7.5), 0.3 M NaCl, 5% glycerol, 0.5 mM TCEP (VWR life sciences) and 1 mM PMSF (Roche). After clearing, the lysate was loaded on a Hi-Trap metal chelating column (GE Healthcare). Bound proteins were eluted with an imidazole gradient. The fusion-tag was cleaved from MAD2L2^WT^, MAD2L2^1*x*M*ut*^ and MAD2L2^3*x*M*ut*^ using PreScission protease. Protein containing fractions were pooled, and concentrated in 10 kDa MWCO concentrator (Merck) and loaded onto a Superdex-75 column (GE Healthcare) in tandem with HiTrap Talon Crude 1 ml column (GE Healthcare) equilibrated with buffer B (10 mM HEPES pH 7.5, 0.15 M NaCl, 5% glycerol and 0.5 mM TCEP) for size-exclusion chromatography (SEC). Fractions containing purified MAD2L2 and SHLD3^28-83^ were concentrated, flash-frozen and stored at −80 °C until use. TRIP13^E253Q^, MAD2L2^1*x*M*ut*^-SHLD3 and MAD2L2^WT/1xMut^-SHLD3-SHLD2^1-95^ complexes were purified as His_6_-EGFP, His-MBP fusion and GST-fusion constructs respectively from insect cell using biGBac Expression system^59^. Bacmid was then produced from DH10-beta cells and subsequently used to transfect Sf9 cells and produce baculovirus. Baculovirus was amplified through three rounds of amplification and used to infect Hi5 cells. Cells infected with the viruses were cultured for 72 h before harvesting. Purification of protein complexes was carried out using above protocol while buffer C (25 mM Tris (pH 8), 0.3 M NaCl, 10% glycerol, 0.5 mM TCEP, 10 mM MgCl_2_ and 1 mM PMSF) was used for His_6_-EGFP-TRIP13 ^E253Q^. Further polishing of MAD2L2 containing complexes prior SEC and after tag cleavage with PreScission protease was carried out by loading onto a cation-exchange (CE) Resource-S column (GE Healthcare) equilibrated in 10 mM HEPES (pH 7.5), 50 mM NaCl, 5% glycerol and 0.5 mM TCEP. Elution was carried out using NaCl gradient. Fractions containing purified His_6_-EGFP-TRIP13^E253Q^, MAD2L2^1*x*M*ut*^-SHLD3 and MAD2L2^WT/1xMut^-SHLD3-SHLD2^1-95^ were concentrated, flash-frozen and stored at −80 °C until use.

### In vitro-binding assays

For MBP-pulldown experiments, 0.25 μM MBP-SHLD3^28-83^ pre-adsorbed on Amylose beads was incubated for indicated time points at 4 °C with eightfold molar excess of GST-MAD2L2^1*x*M*ut*^/MAD2L2^WT^/MAD2L2^1*x*M*ut*^/MAD2L2^3*x*M*ut*^ in buffer B. After two washing steps of 0.5 ml each with buffer B, complexes immobilized on beads were analyzed by 12% SDS-PAGE. The gels were digitized and quantified using Fiji^60^. For TRIP13 pulldown experiments, purified proteins were mixed at 6 µM in PD-buffer containing 25 mM Tris (pH 8), 0.3 M NaCl, 10% glycerol, 10 mM MgCl_2_, 0.5 mM TCEP, 0.01% Triton-X and 3 mM ATP. After 5-min incubation period, glutathione agarose or amylose beads were added. Beads were washed twice with PD-buffer + 0.1 mM ATP and analyzed by 12% SDS-PAGE.

### Size-exclusion chromatography

SEC runs were performed on Superdex-200 column (GE Healthcare) equilibrated with buffer C + 0.1 mM ATP. Prior run proteins were incubated for 2 h on ice in buffer C + 2 mM ATP. For GST induced REV7 dimerization profiles, SEC runs were performed on Superdex-200 column equilibrated with buffer B. Fractions collected from SEC runs were analyzed by 12% SDS-PAGE.

### Statistics and reproducibility

Statistical analyses were performed with GraphPad Prism using the tests as indicated in the respective figure legends. Significance; ns, not significant (*p* ≥ 0.05), *p < 0.05, **p < 0.01, ***p < 0.001, ****p < 0.0001. Unless indicated otherwise, all immunoblots are representative of at least two independent experiments, with uncropped blots shown in the Source data file.

### Reporting summary

Further information on research design is available in the Nature Research Reporting Summary linked to this article.

## Supplementary information

Supplementary Information

Reporting Summary

## Data Availability

The data that support this study are available from the corresponding author upon reasonable request. The mass-spectrometry proteomics data generated in this study have been deposited to the ProteomeXchange Consortium via the PRIDE partner repository with the dataset identifier PXD026912. Source data are provided with this paper.

## Source data

Source Data
