# Supplementary material for: MAD2L2 dimerization and TRIP13 control shieldin activity in DNA repair
Source: Nat Commun. 2021 Sep 14;12:5421. doi: 10.1038/s41467-021-25724-y (PMC8440562; doi:10.1038/s41467-021-25724-y)
Supplement: Supplementary file 1 — Supplementary Information [file 41467_2021_25724_MOESM1_ESM.pdf]

Supplementary Information

MAD2L2 dimerization and TRIP13 control shieldin activity in DNA repair

Inge de Krijger[1], Bastian Föhr[2,#], Santiago Hernández Pérez[1,#], Estelle Vincendeau[3,4,#], Judit Serrat[1,#], Alexander Marc Thouin[1,#], Vivek Susvirkar[2], Chloé Lescale[3], Inés Paniagua[1], Liesbeth Hoekman[5], Simranjeet Kaur[2], Maarten Altelaar[5,6], Ludovic Deriano[3], Alex C. Faesen[2], Jacqueline J. L. Jacobs[1,*]

This file contains:

- Supplementary Fig. 1
- Supplementary Fig. 2
- Supplementary Fig. 3
- Supplementary Fig. 4
- Supplementary Fig. 5
- Supplementary Fig. 6
- Supplementary Fig. 7
- Supplementary Fig. 8
- Supplementary Table 1

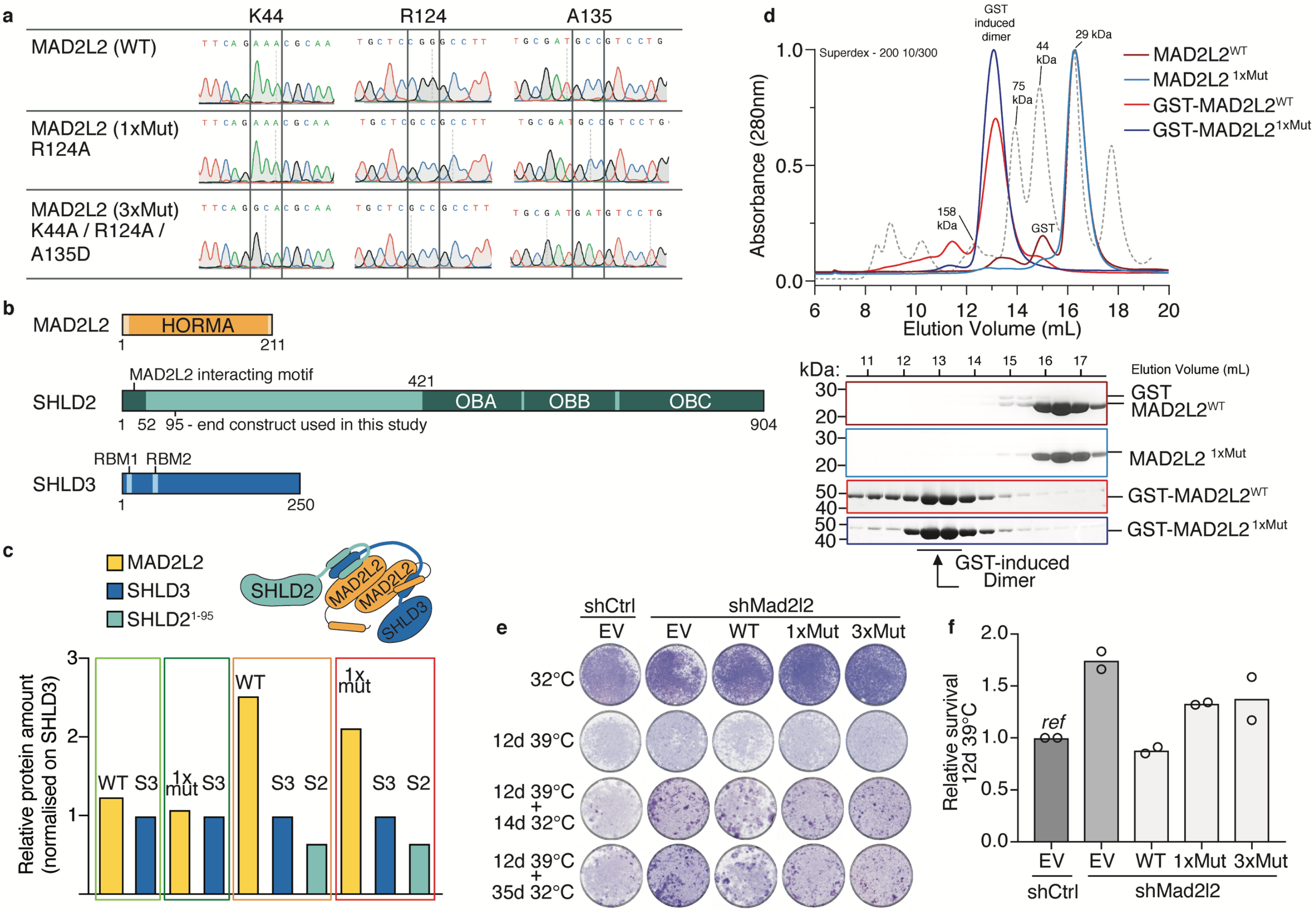

Supplementary Figure 1: Residues at the MAD2L2 dimerization surface mediate MAD2l2-dimerization and shieldin complex formation. **a**, Sequencing files showing the mutations introduced in the MAD2L2 CDS. **b,** Schematic representation of MAD2L2, SHLD3 and SHLD2 recombinant proteins used in *in vitro* experiments in Fig. 1, 3 and Supplementary Fig. 1. **c,** Quantification of Fig. 1d. Protein amount normalized on SHLD3. **d,** SEC profiles of MAD2L2$^{WT/1xMut}$ and N-terminal GST fusion MAD2L2 $^{WT/1xMut}$ on Superdex-200 column equilibrated in buffer B. **e,** Independent biological duplicate of experiment shown in Fig. 2e. **f,** Quantification of the survival assay shown in Fig. 2e and **e**, crystal violet extraction of survival after 12 days at 39°C and plotted relative to control condition. Bars represent the mean, dots indicate n=2 individual experiments. Source data are provided as a Source Data file.

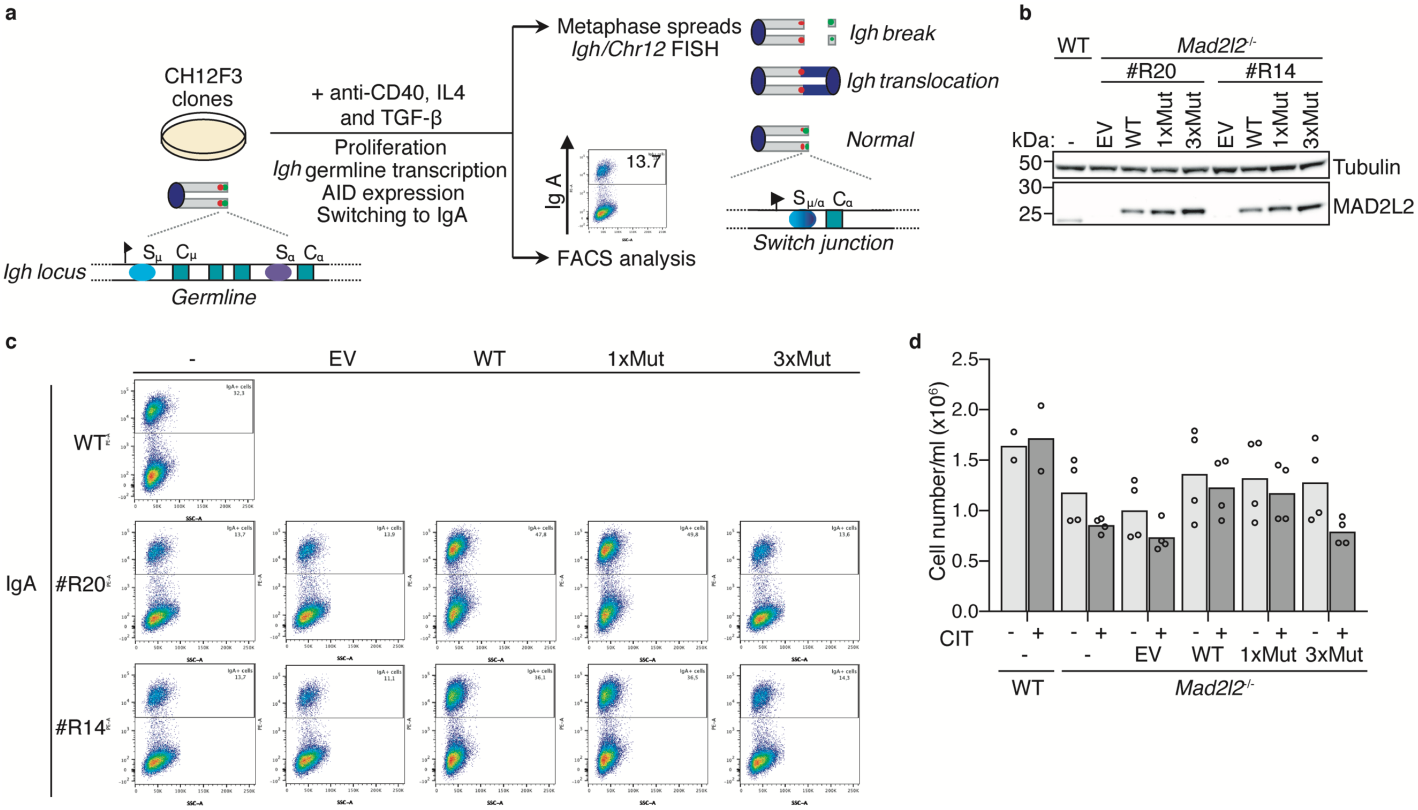

Supplementary Figure 2: MAD2L2 dimerization is essential for MAD2L2-dependent class switch recombination. a, Schematic overview of the experimental setup used in Fig. 2f-h. CSR and chromosomal instability in murine CH12 B cells (germline configuration with Cμ transcription) induced to express AID and undergo CSR to IgA (switch configuration with Cα transcription) on addition of anti-CD40, interleukin-4 (IL-4) and transforming growth factor beta (TGF-β). CSR levels were measured as percent of IgA positive cells after 72h cytokine stimulation, and DNA fluorescence in situ hybridization (FISH) was performed using a chromosome 12-specific paint (grey chromosome) and *Igh* locus-specific probes (red and green spots) for the measurement of chromosomal instability at the *Igh* locus upon induction of CSR. **b**, Immunoblot showing *Mad2l2*$^{-/-}$ cell clones (#R20 and #R14) complemented with empty vector (EV), WT MAD2L2, 1xMut and 3xMut, as used in Fig. 2f-h. **c,** Representative flow cytometry profiles showing the percentage of IgA^{+} cells for indicated WT and *Mad2l2*$^{-/-}$ clones (#R20 and #R14) after 4 days stimulation with anti-CD40, IL-4 and TGF-β. **d,** WT, *Mad2l2*$^{-/-}$ cells were plated at 50,000 cells/ml and counted after 4 days stimulation with anti-CD40, IL4, and TGF-β. Bars represent means, dots represent n=2 independent experiments with one WT clone and two *Mad2l2*$^{-/-}$ clones. Source data are provided as a Source Data file.

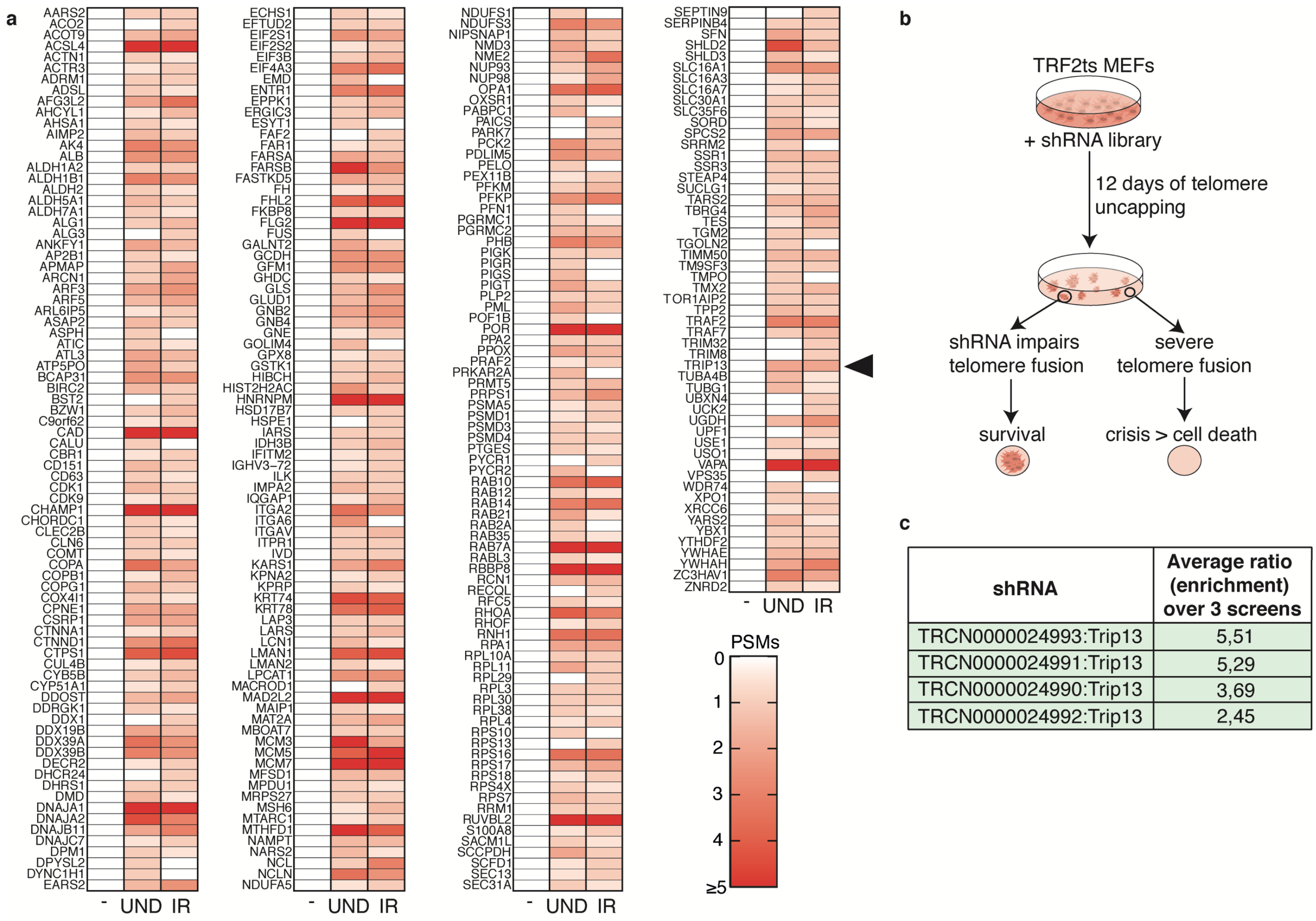

shRNA	Average ratio (enrichment) over 3 screens
TRCN0000024993:Trip13	5,51
TRCN0000024991:Trip13	5,29
TRCN0000024990:Trip13	3,69
TRCN0000024992:Trip13	2,45

Supplementary Figure 3: MAD2L2 pulldown and functional genetic screening identified TRIP13 as an important protein in DNA repair. a, Heatmap representation of MAD2L2 interacting proteins upon pulldown of GFP-tagged MAD2L2 or GFP vector control (Control) followed by mass spectrometry analysis. For the pulldown, HeLa cells were depleted for endogenous MAD2L2 and complemented with either a GFP-tagged RNAi-resistant MAD2L2 or an GFP-expressing empty vector (-). Cells were left untreated (UND) or irradiated with 25Gy followed by 3h recovery (IR). The spectral count results of two biological replicate experiments were averaged and represented with a color code. Only proteins with at least one count (average of two replicates) in one of the two conditions and zero counts in the control condition are shown. The color code ranges from white (0 PSM(s)) to red (≥5 PSM(s)), genes are sorted alphabetically. **b,** Schematic representation of a functional genetic screen ('TIGIR' screen) for factors promoting genomic crisis upon TRF2 inactivation-induced telomere uncapping in temperature-sensitive TRF2 (TRF2ts) MEFs[1]. **c,** The setup described in **b** and previously reported[1], yielded independent shRNAs against TRIP13 that were enriched >1.5-fold in at least two out of three screens with a small DDR-focused shRNA library. Average ratios of enrichment over all three screens are shown and reflect shRNA abundance after 12 days of telomere uncapping at 39°C followed by 4 days recovery at 32°C versus shRNA abundance after growth for 4 days at 32°C. Enrichment of shRNAs indicates that these shRNAs enable cells to survive by avoiding genomic crisis caused by prolonged uncapping of telomeres and associated massive chromosome end-to-end fusion[1]. Source data are provided as a Source data file.

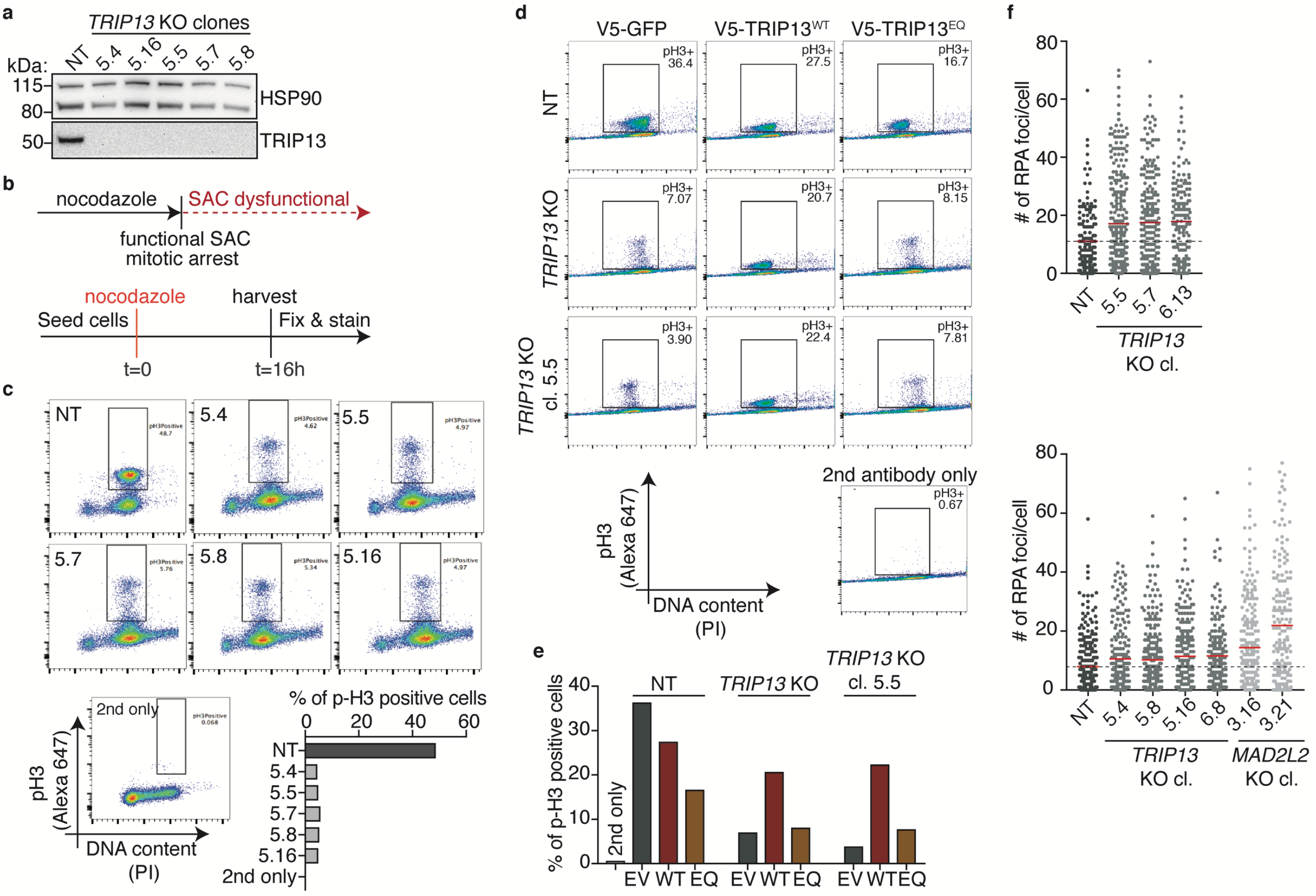

Supplementary Figure 4: *TRIP13* KO clones lack a functional SAC. a, Immunoblot for TRIP13 in control U2OS cells (NT, non-targeting cells) or multiple individual *TRIP13* KO U2OS clones, of which 5.5, 5.7, 5.8, 5.16 were pooled to obtain a polyclonal full KO cell line used in Fig. 4g, Suppl. Fig. 5d, e ('*TRIP13* KO'). Cells were harvested 3h post 25Gy. HSP90 serves as loading control. **b**, Schematic representation of SAC-activation upon nocodazole treatment and experimental setup in **c-e**. **c**, pH3 staining in *TRIP13* KO U2OS cells (clones 5.4-5.16) or NT cells shown in **a.** The quantification of pH3+ cells is shown below. Control U2OS cells efficiently arrest in mitosis upon disrupting the mitotic spindle with nocodazole, seen as increased phosphorylation of histone $H3^{Ser10}$, marking cells in mitosis. As expected[2], *TRIP13* KO cells continued cell cycle progression in the presence of nocodazole, indicating a dysfunctional SAC. **d,** pH3 staining in *TRIP13* KO U2OS cells (*TRIP13* KO, polyclonal full KO; *TRIP13* KO cl. 5.5, single *TRIP13* KO clone) or NT cells complemented with the indicated empty vector (V5-GFP) or TRIP13 constructs (WT or ATPase-dead EQ mutant). Exogenous expression of WT TRIP13 restored the ability of *TRIP13* KO cells to arrest in mitosis, while the EQ mutant did not**. e**, Quantification of pH3+ cells as shown in **d**. **f**, quantification of RPA foci in NT cells, multiple individual *TRIP13* KO clones or two individual partial *MAD2L2* KO clones in U2OS cells. Clones indicated by '5' are made with *TRIP13* sgRNA#1, and clones indicated by '6' are made with sgRNA#2. A minimum of 153 cells were analyzed per clone. Source data are provided as a Source Data file.

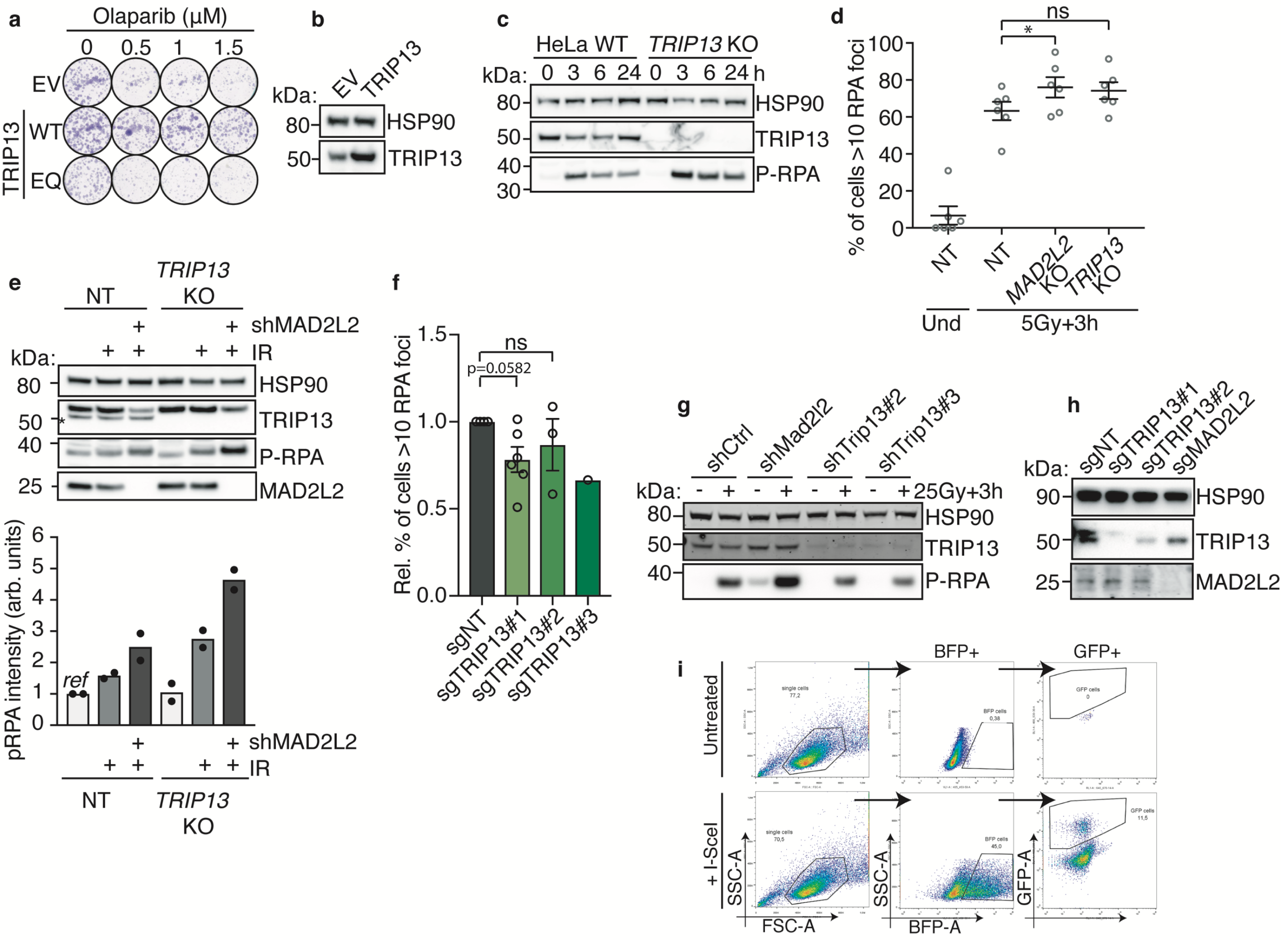

Supplementary Figure 5: TRIP13 impacts on end-resection control. a, Plates belonging to the survival curve in Fig. 4d. **b**, Immunoblot of TRIP13 levels in cells used in Fig. 4e. **c,** Immunoblot of iCas9 expressing parental HeLa wild-type (WT) or *TRIP13* KO (sgRNA#2) cells irradiated with 25Gy and harvested at different timepoints after irradiation as indicated. **d**, Quantification of RPA foci 3h post IR (5Gy) in cells used in Fig. 4g. Dots represent individual experiments, showing the % of cells with >10 RPA foci. Per replicate 175-420 cells were analyzed for each condition, except for undamaged cells (85-155 cells per replicate) (n=6, graphs represent mean±s.e.m., statistical analysis was performed on IR-treated samples, one-way ANOVA with Dunnett's multiple comparisons test, ns *p*=0.0703; **p*=0.0384). **e,** Immunoblot of control (NT) or *TRIP13* KO U2OS cells transduced as indicated and treated with IR or left undamaged. (*) star indicates TRIP13 band. Quantification of p-RPA signal below (n=2, replicates indicated as dots, arb. units, arbitrary units). **f,** Data as in Fig. 4i with 2 additional experiments for sgNT, sgTRIP13#1 and sgTRIP13#2 not included in Fig. 4i due to lack of an sgMAD2L2 positive control (mean±s.e.m., n=6 for sgNT/sgTRIP13#1, n=3 for sgTRIP13#2, one way ANOVA with Dunnett's multiple comparisons test, except sgTRIP13#3 for which n=1, ns *p*=0.0582 (sgNT vs. sgTRIP13#1) and *p*=0.4075 (sgNT vs. sgTRIP13#2). **g,** Immunoblot of TRF2ts MEFs depleted for MAD2L2 or TRIP13 using two independent shRNAs, untreated or harvested 3h post IR (representative of n=2 independent experiments). **h,** Immunoblot of U2OS DIvA cells transduced with sgRNAs as indicated and used in Fig. 4j. **i.** Gating strategy for analysis of DR-GFP U2OS reporter cells, used in Fig. 4e. Significance; ns, not significant (*p*≥0.05), **p*<0.05. Source data are provided as a Source Data file.

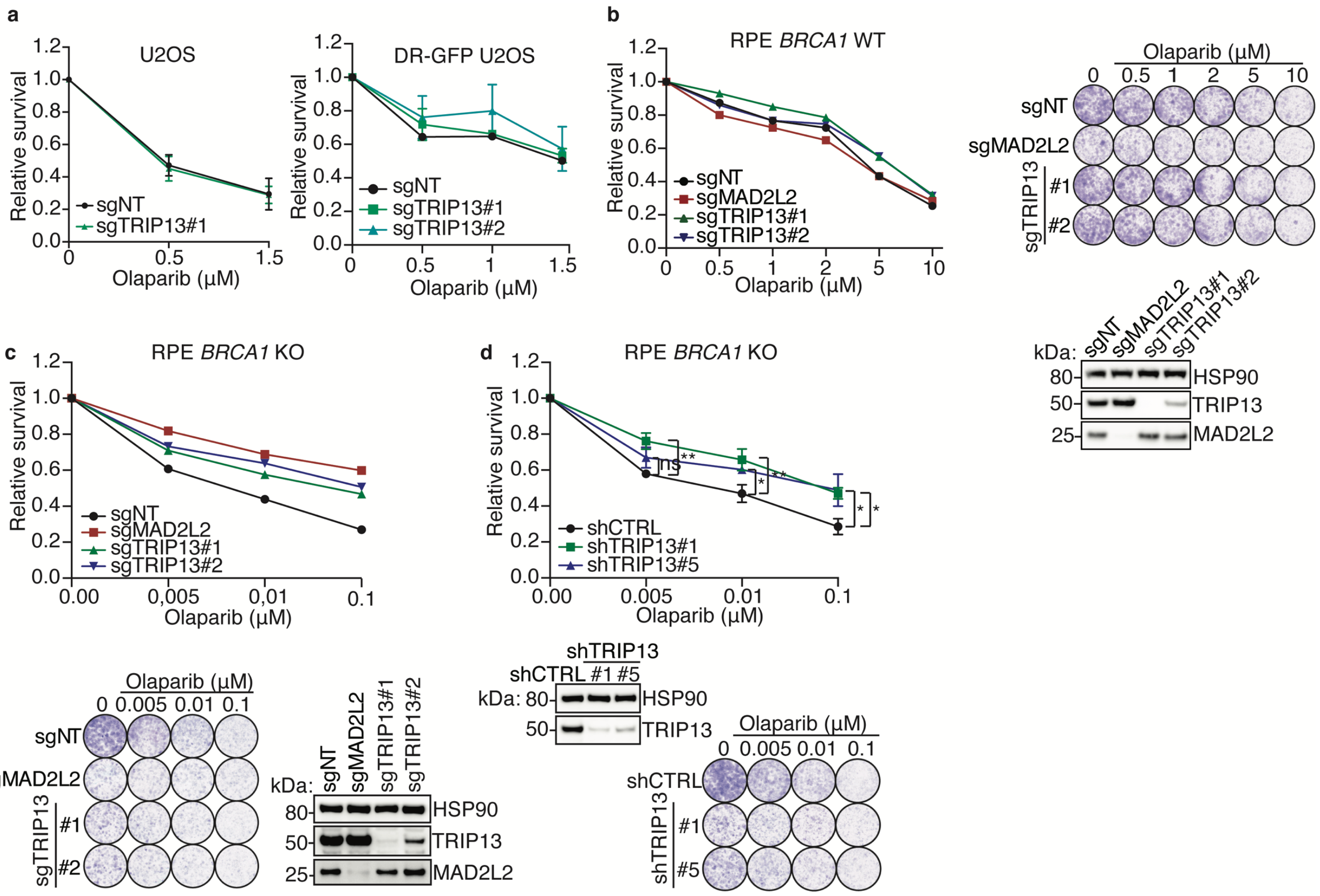

Supplementary Figure 6: TRIP13 affects olaparib sensitivity. a, Survival curves of U2OS and DR-GFP U2OS cells transduced as indicated and treated with indicated concentrations of olaparib. Graph represents mean±s.e.m. of n=4 (U2OS) or n=5 (DR-GFP U2OS) biologically independent experiments. **b,** Survival curve of RPE cells transduced as indicated and treated with the indicated concentrations of olaparib. Graphs represent mean of n=2 independent experiments. Representative plates and immunoblot are shown on the right. **c,** Survival curve in *BRCA1*-deficient RPE cells transduced as indicated. Representative plates and immunoblot are shown below, graph represents mean of n=2 independent experiments**. d,** Survival curve in *BRCA1*-deficient RPE cells transduced with indicated shRNAs. Graphs represent mean±s.d. of n=3 biologically independent experiments, statistical analysis was done for each olaparib concentration by one-way ANOVA with Dunnett's multiple comparisons test (ns p=0.0749 and **p=0.0039 (0.005µM); *p=0.0199 and **p=0.0041 (0.01µM); *p=0.0168 (shCTRL vs. shTRIP13#1, 0.1µM) and p=0.0109 (shCTRL vs. shTRIP13#5, 0.1µM)). Plates and immunoblot showing the depletion of TRIP13 are shown below. Significance; ns, not significant (p≥0.05), *p<0.05, **p<0.01. Source data are provided as a Source Data file.

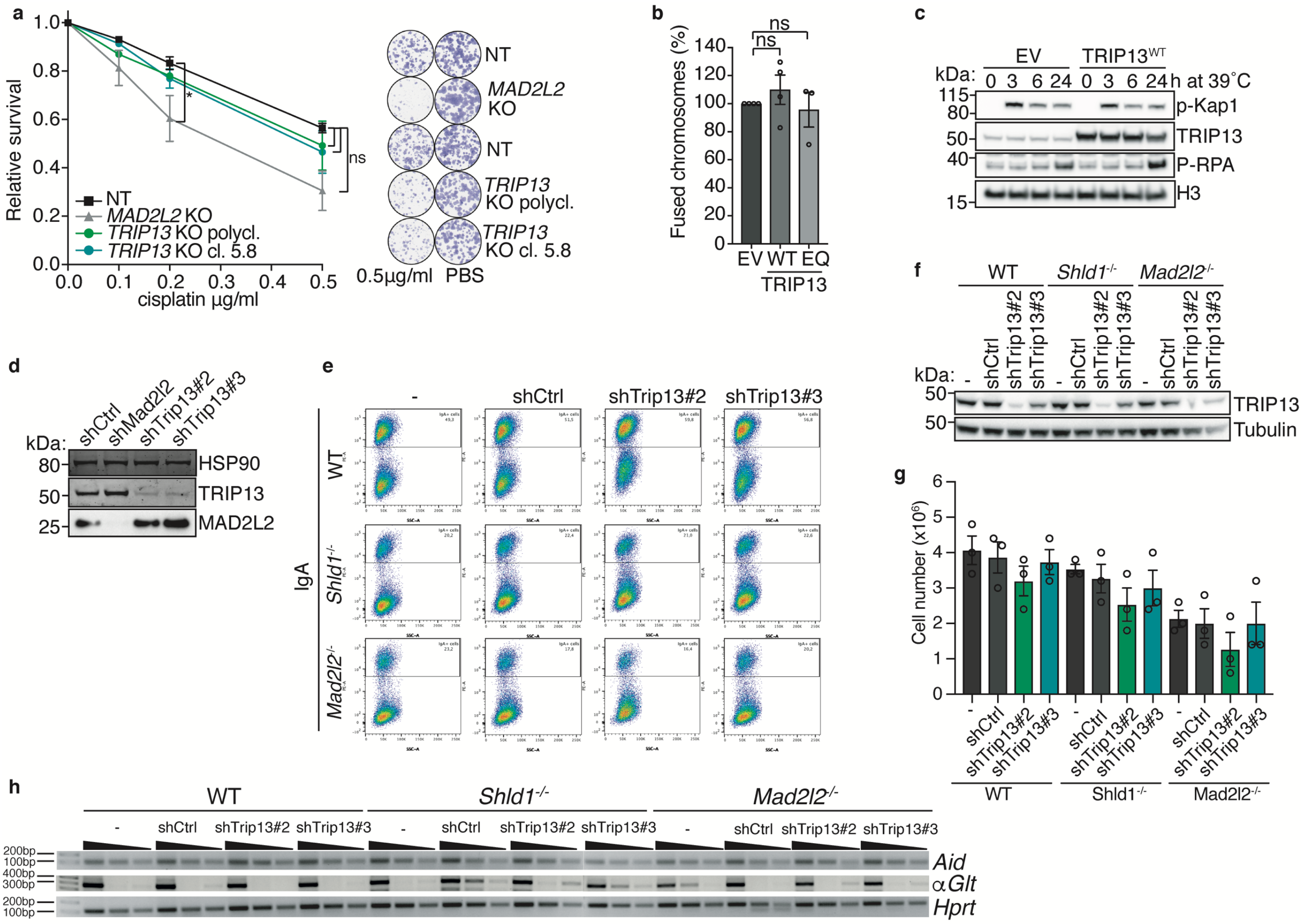

Supplementary Figure 7: TRIP13 affects DNA repair. a, Survival curve of NT control U2OS cells, a *MAD2L2* KO U2OS clone and *TRIP13* KO U2OS cells (polyclonal and single 5.8 KO clone) treated with indicated doses of cisplatin. For quantification, the two NT-control lines were averaged. Representative plates are shown on the right (graph represents mean±s.e.m of n=3 biologically independent experiments, one-way ANOVA with Dunnett's multiple comparisons test, **p*=0.0400; ns *p*=0.1154 (NT vs. *MAD2L2* KO), *p*=0.8528 (NT vs. *TRIP13* KO) and *p*=0.7125 (NT vs. *TRIP13 KO* cl5.8)). **b,** Quantification of chromosome fusions in TRF2ts MEFs transduced as indicated and cultured at the non-permissive temperature of 39°C for 24h to induce telomere uncapping. Values are relative to empty vector (EV) transduced cells. Date points represent individual experiments (n=4, except EQ for which n=3, mean±s.e.m., >1,500 chromosomes counted per condition per experiment, one-way ANOVA with Dunnett's multiple comparisons test, ns *p*=0.6250 (EV vs. WT) and *p*=0.9219 (EV vs. EQ)). **c**, TRF2ts MEFs transduced as indicated and cultured at 39°C for the indicated timepoints to induce telomere uncapping. **d,** Representative immunoblot of TRF2ts MEFs transduced as indicated and used in Fig. 5a. **e,** Representative flow cytometry profiles showing the percentage of IgA^+ cells for indicated WT, $Shld1^{-/-}$ and $Mad2l2^{-/-}$ cells after 4 days stimulation with anti-CD40, IL-4 and TGF-β. **f**, Immunoblot showing TRIP13 knock-down in WT, $Shld1^{-/-}$ and $Mad2l2^{-/-}$ cells as used in Fig. 5b. **g**, WT, $Shld1^{-/-}$ and $Mad2l2^{-/-}$ cells were plated at 50,000 cells/ml and counted after 4 days stimulation with anti-CD40, IL4, and TGF-β. Bars represent means ± s.e.m.; n=3 independent experiments. **h**, *Igh*, α germ-line transcripts (αGLT) and *Aid* mRNA were quantified by semi-quantitative RT–PCR using 2.5-fold serial dilutions of cDNA made from WT, $Shld1^{-/-}$ and $Mad2l2^{-/-}$ cells after 4 days stimulation with anti-CD40, IL4, and TGF-β. *Hprt* was used as a control for transcript expression. Significance; ns, not significant ($p \geq 0.05$), **p*<0.05. Source data are provided as a Source Data file.

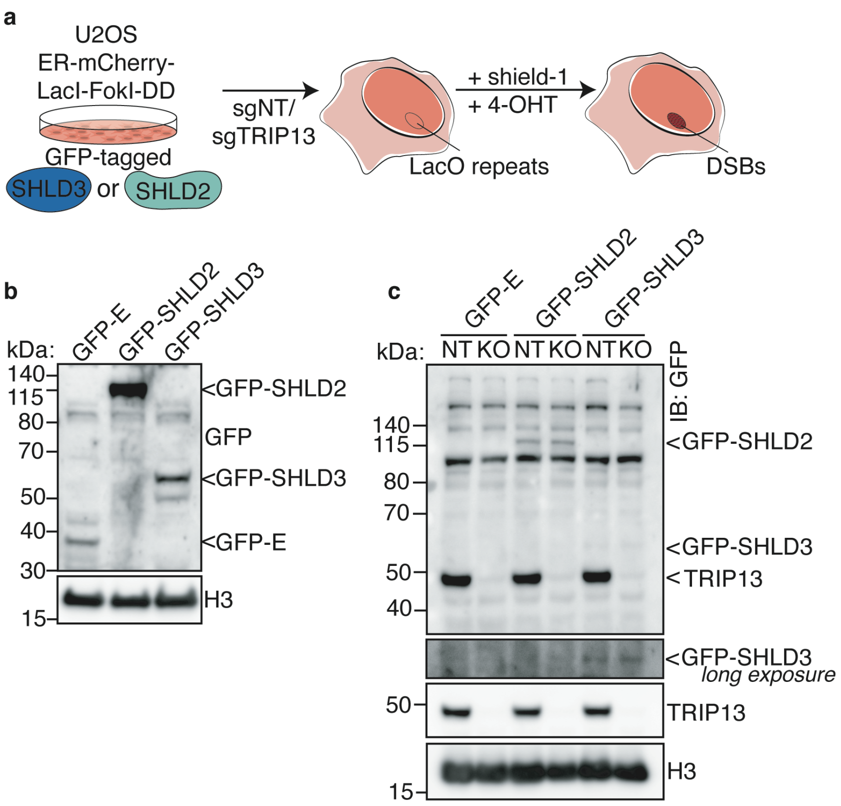

Supplementary Figure 8: TRIP13 controls the assembly of SHLD2/3 at DNA breaks. a, Schematic representation of U2OS ER-mCherry-LacI-FokI-DD system used in Fig. 6b-e. **b,** Immunoblot of Phoenix cells transfected with the indicated constructs used in Fig. 6b-e. H3 serves as loading control. **c**, Representative immunoblot of cells used in Fig. 6b-e. U2OS ER-mCherry-LacI-FokI-DD cells transduced with GFP-expressing constructs as indicated and depleted for TRIP13. H3 serves as loading control. Source data are provided as a Source Data file.

Supplementary Table 1

Primer list

#	Type	Primer	Sequence (5’ - 3’)
1.	Mutagenesis	R124A_Fw	CATGTGGAGCAGCTGCTCGCCGCCTTCATCCTGAAGATC
2.	Mutagenesis	R124A_Rev	GATCTTCAGGATGAAGGCGGCGAGCAGCTGCTCCACATG
3.	Mutagenesis	K44A_Fw	CGTGGGGATTTTTCAGGCACGCAAGAAGTACAAC
4.	Mutagenesis	K44A_Rev	GTTGTACTTCTTGCGTGCCTGAAAAATCCCCACG
5.	Mutagenesis	A135D_Fw	CAGCGTGTGCGATGATGTCCTGGACCACAAC
6.	Mutagenesis	A135D_Rev	GTTGTGGTCCAGGACATCATCGCACACGCTG
7.	Mutagenesis	E253Q_Fw	CGTGCTGATTGATCAGGTGGAGAGTCTC
8.	Mutagenesis	E253Q_Rev	GAGACTCTCCACCTGATCAATCAGCACG
9.	PCR end-resection	Fw	ACCATGAACGTGTTCCGAAT
10.	PCR end-resection	Rev	GAGCTCCGCAAAGTTTCAAG
11.	RT-PCR analysis CSR	Hprt_Fw	CTGGTGAAAAGGACCTCTCG
12.	RT-PCR analysis CSR	Hprt_Rev	TGAAGTACTCATTATAGTCAAGGGCA
13.	RT-PCR analysis CSR	Aid_Fw	GAAAGTCACGCTGGAGACCG
14.	RT-PCR analysis CSR	Aid_Rev	TCTCATGCCGTCCCTTGG
15.	RT-PCR analysis CSR	αGLT_Fw	GACATGATCACAGGCACAGG
16.	RT-PCR analysis CSR	αGLT_Rev	TTCCCCAGGTCACATTCATCGT

References

1. Boersma, V. *et al.* MAD2L2 controls DNA repair at telomeres and DNA breaks by inhibiting 5' end resection. *Nature* **521**, 537-540 (2015).
2. Ma, H.T. & Poon, R.Y.C. TRIP13 Regulates Both the Activation and Inactivation of the Spindle-Assembly Checkpoint. *Cell Rep* **14**, 1086-1099 (2016).
