# Supplementary material for: MAD2L2 dimerization and TRIP13 control shieldin activity in DNA repair
Source: Nat Commun. 2021 Sep 14;12:5421. doi: 10.1038/s41467-021-25724-y (PMC8440562; doi:10.1038/s41467-021-25724-y)
Supplement: Supplementary file 2 — Reporting Summary [file 41467_2021_25724_MOESM2_ESM.pdf]

nature research

Corresponding author(s): J.J.L. Jacobs

Last updated by author(s): Jul 2, 2021

Reporting Summary

Nature Research wishes to improve the reproducibility of the work that we publish. This form provides structure for consistency and transparency in reporting. For further information on Nature Research policies, see our Editorial Policies and the Editorial Policy Checklist.

Statistics

For all statistical analyses, confirm that the following items are present in the figure legend, table legend, main text, or Methods section.

n/a	Confirmed	
☐	☒	The exact sample size (*n*) for each experimental group/condition, given as a discrete number and unit of measurement
☐	☒	A statement on whether measurements were taken from distinct samples or whether the same sample was measured repeatedly
☐	☒	The statistical test(s) used AND whether they are one- or two-sided<br>*Only common tests should be described solely by name; describe more complex techniques in the Methods section.*
☒	☐	A description of all covariates tested
☐	☒	A description of any assumptions or corrections, such as tests of normality and adjustment for multiple comparisons
☐	☒	A full description of the statistical parameters including central tendency (e.g. means) or other basic estimates (e.g. regression coefficient) AND variation (e.g. standard deviation) or associated estimates of uncertainty (e.g. confidence intervals)
☐	☒	For null hypothesis testing, the test statistic (e.g. *F*, *t*, *r*) with confidence intervals, effect sizes, degrees of freedom and *P* value noted<br>*Give P values as exact values whenever suitable.*
☒	☐	For Bayesian analysis, information on the choice of priors and Markov chain Monte Carlo settings
☒	☐	For hierarchical and complex designs, identification of the appropriate level for tests and full reporting of outcomes
☒	☐	Estimates of effect sizes (e.g. Cohen's *d*, Pearson's *r*), indicating how they were calculated

Our web collection on statistics for biologists contains articles on many of the points above.

Software and code

Policy information about availability of computer code

Data collection	1. Syngene G:BOX (Syngene)<br>2. Odyssey Infrared imager (LI-COR)<br>3. Leica SP5 (Leica Microsystems)<br>4. LSR II Flow Cytometer (BD Biosciences)<br>5. Metafer4/MSearch (Metasystems) on AxioImagerZ2 microscope (Carl Zeiss)<br>6. Infinite M200pro (TECAN)<br>7. Fortessa analyser (BD Biosciences)<br>8. Orbitrap Fusion Tribrid mass spectrometer with Proxeon nLC1000 system (Thermo Scientific)<br>9. Attune TM NxT Acoustic Focusing Cytometer (Thermo Fisher Scientific)
Data analysis	1. Syngene G:BOX (version 1.6.9.0)<br>2. Fiji/ImageJ (version 1.52p)<br>3. LAS-AF (version 2.7.4)<br>4. FlowJo (version 10)<br>5. GraphPad Prism (version 7.0c)<br>6. Proteome Discoverer (PD, version 2.3.0.523, Thermo Scientific)<br>7. R (version 4.0.2)<br>8. Perseus (version 1.6.14.0)<br>9. Microsoft Excel (version 16.16.27)

For manuscripts utilizing custom algorithms or software that are central to the research but not yet described in published literature, software must be made available to editors and reviewers. We strongly encourage code deposition in a community repository (e.g. GitHub). See the Nature Research guidelines for submitting code & software for further information.

Data

Policy information about availability of data

All manuscripts must include a data availability statement. This statement should provide the following information, where applicable:

- Accession codes, unique identifiers, or web links for publicly available datasets
- A list of figures that have associated raw data
- A description of any restrictions on data availability

Source data for all figures are provided in the Source data file. This includes all uncropped blots, gels, and data shown in graphs throughout the manuscript, including the Supplementary Figures. The mass spectrometry proteomics data generated in this study, and shown in Figure 6a and Supplementary Figure 3a, have been deposited to the ProteomeXchange Consortium with the dataset identifier PXD026912.
All data are available from the authors upon reasonable request.

Field-specific reporting

Please select the one below that is the best fit for your research. If you are not sure, read the appropriate sections before making your selection.

- [x] Life sciences
- [ ] Behavioural & social sciences
- [ ] Ecological, evolutionary & environmental sciences

For a reference copy of the document with all sections, see nature.com/documents/nr-reporting-summary-flat.pdf

Life sciences study design

All studies must disclose on these points even when the disclosure is negative.

Sample size	No sample-size calculations were performed. The number of biological replicates is mentioned in the figure legends and the corresponding data point for each individual biological replicate are shown in the graphs. We included information on the (range in the) number of cells used for IF-analysis in the figure legends.<br>1. For metaphase spread analysis to address chromosomal fusions, we analyzed at least 30 metaphase spreads per condition per experiment, to get to a total of at least 1500 chromosomes per condition within each biological replicate. Analysis of >1000 chromosomes per condition and replicate is sufficient to obtain a reliable mean.<br>2. For immunofluorescence experiments, we aimed to acquire a minimum of 100 cells per condition per experiment in treated condition. For untreated cells (serving as negative control), we analyzed a minimum of 85 individual cells per condition per experiment. Analysis of >100 cells for treated conditions was sufficient to obtain a representative measure (reliable mean) of the number of foci of an experimental condition. Figure legends of the individual experiments indicate the number of cells analyzed.<br>3. Sensitivity assays were seeded as technical duplicates for each condition within one biological duplicate, to average out the variation between technical replicates.
Data exclusions	No samples or data points were excluded from individual replicate experiments. In the rare event that internal negative or positive controls within an individual experiment did not perform as expected, this entire individual experiment (with all samples) was discarded.
Replication	Most experiments shown contain internal controls that have been validated before. Experiments were performed at least in duplicate, but mostly in triplicate or more to assess the reproducibility (where duplicates, they mostly represent control blots). Graphs are represented as dot plots showing the spread among the replicates of the individual experiments. Standard errors are included in graphs that contain more than two replicates and that indicate the variation between replicates. Number of biological replicates for each experiment are indicated in the respective figure legends. For the experiment shown in Suppl. Figure 4f, multiple individual clones were analyzed, and dots represent individual cells analyzed within that clone.
Randomization	No particular method of sample randomization was used, though no specific order of processing samples was maintained between experiments. Experiments were performed with cell lines that were seeded randomly for different treatments.
Blinding	Genotypes were blinded after harvesting and prior to manual scoring of the samples for 2 out of 5 experiments in Figure 5a, and 3 out of 4 experiments in Supplemental Figure 7b.<br>For many other approaches used in the manuscript, including western blots and IPs, blinding was not feasible.

Reporting for specific materials, systems and methods

We require information from authors about some types of materials, experimental systems and methods used in many studies. Here, indicate whether each material, system or method listed is relevant to your study. If you are not sure if a list item applies to your research, read the appropriate section before selecting a response.

Materials & experimental systems

n/a	Involved in the study
[ ]	[x] Antibodies
[ ]	[x] Eukaryotic cell lines
[x]	[ ] Palaeontology and archaeology
[x]	[ ] Animals and other organisms
[x]	[ ] Human research participants
[x]	[ ] Clinical data
[x]	[ ] Dual use research of concern

Methods

n/a	Involved in the study
[x]	[ ] ChIP-seq
[ ]	[x] Flow cytometry
[x]	[ ] MRI-based neuroimaging

Antibodies

Antibodies used

For an overview of antibodies used, see Material and Methods section.
Primary antibodies:
1. HSP90, sc7947, Santa-Cruz, 1:500
2. MAD2L2, sc135977, Santa-Cruz, 1:500
3. MAD2L2, 12683-1-AP, ProteinTech, 1:500
4. V5, R96025, Invitrogen, 1:500
5. GFP, A11122, Life Technologies, 1:1,000
6. TRIP13, ab128153, Abcam, 1:1,000
7. p-RPA, RPA2 pS4/pS8, NBP1-23017, Novus Biologicals, 1:500
8. Phospho-RPA32 S4/S8, A300-245A, Bethyl, 1:1,000
9. H3, ab1791, Abcam, 1:1,000
10. H2B, 07-371, Millipore, 1:1,000
11. Tubulin, T6557, Sigma, 1 :10,000
12. p-Kap1, S824, A300-767A, Bethyl, 1:1,000
13. RPA, RPA34-20, NA18, Calbiochem, 1:500
14. p-H3 Ser10, 06-570, Millipore, 1:500
15. IgA-PE, 12-4204-82, eBiosciences, 1:200
16. Flag-M2 Magnetic beads, M8823, Sigma-Aldrich

Secondary antibodies:
1. Alexa Fluor 488 goat anti-mouse IgG A11029, Invitrogen, 1:500
2. Alexa Fluor 488 goat anti-rabbit IgG A11008, Invitrogen, 1:500
3. Alexa Fluor 568 goat anti-mouse IgG A11031, Invitrogen, 1:500
4. Alexa Fluor 568 goat anti-rabbit IgG A211011, Invitrogen, 1:500
5. Alexa Fluor 647 goat anti-rabbit IgG A21246, Invitrogen, 1:500
6. Goat anti-rabbit IgG HRP, G21234, Invitrogen, 1:7,500
7. Goat anti-mouse IgG HRP, G21040, Invitrogen, 1:7,500
8. IRDye800CW Goat anti-mouse IgG, 926-32210, LI-COR, 1:10,000
9. IRDye800CW Goat anti-rabbit IgG, 926-32211, LI-COR, 1:10,000
10. IRDye680 Goat anti-mouse IgG, 926-32220, LI-COR, 1:10,000
11. IRDye680 Goat anti-rabbit IgG, 926-32221, LI-COR, 1:10,000

Validation

Commercially available antibodies were validated by the manufacturer/supplier.

The following antibodies were additionally validated by us in different applications as stated below:
- Knockdown/knockout cells: MAD2L2 (sc135977, Santa Cruz), TRIP13 (ab128153, Abcam), MAD2L2 (12683-1-AP, ProteinTech).
- Co-IP experiments: V5 (R96025, Invitrogen), GFP (A11122, Life Technologies), MAD2L2 (sc135977, Santa Cruz), Flag-M2 Magnetic beads (M8832, Sigma-Aldrich)
- Western blot analysis to detect DNA damage-induced phosphorylation: p-RPA (RPA2 pS4/pS8, NBP1-23017, Novus Biologicals), Phospho-RPA32 S4/8 (A300-245A, Bethyl), p-Kap1 S824 (A300-767A, Bethyl)
- DNA damaged-induced foci: GFP (A11122, Life Technologies), RPA (RPA34-20, NA18, Calbiochem)
- Flow cytometry: p-H3 Ser10 (06-570, Millipore), IgA-PE (12-4204-82, eBiosciences)

The following antibodies are commonly used loading controls: HSP90 (sc7947, Santa Cruz), H2B (07-371, Millipore), Tubulin (T6557, Sigma), H3 (ab1791, Abcam)

Eukaryotic cell lines

Policy information about cell lines

Cell line source(s)

1. HeLa, U2OS, 293Ts and Phoenix originate from ATCC.
2. RPE and BRCA1-/- RPE were a gift from D. Durocher and are described before (Zimmerman M. et al, Nature, 2018).

3. TRF2ts MEFs are described before (Konishi et al, Genes & Development, 2008, Peuscher et al, Nature cell biology, 2011).
4. U2OS ER-mCherry-LacI-Fok1-DD cells were a gift from R. Greenberg and described before (Tang J. et al, Nat Struct Mol Biol, 2013)
5. DR-GFP U2OS cells were a gift from J. Stark and are described before (Gunn et al, Methods Mol Biol, 2012).
6. AID-DIvA cells were a gift from G. Legube and are described before (Aymard et al, Nat Struct Mol Biol, 2014)
7. HeLa cells expressing dox-inducible GFP-tagged TRIP13(EQ) were a gift from G. Vader
8. TRIP13 KO HeLa cells were generated by B. de Wolf and G. Kops

Authentication	None of the cell lines used were authenticated by us.
Mycoplasma contamination	All cell lines are routinely tested negative for mycoplasma contamination.
Commonly misidentified lines (See ICLAC register)	No commonly misidentified cell lines were used in this study.

Flow Cytometry

Plots

Confirm that:

- [x] The axis labels state the marker and fluorochrome used (e.g. CD4-FITC).
- [x] The axis scales are clearly visible. Include numbers along axes only for bottom left plot of group (a 'group' is an analysis of identical markers).
- [x] All plots are contour plots with outliers or pseudocolor plots.
- [x] A numerical value for number of cells or percentage (with statistics) is provided.

Methodology

Sample preparation	Sample preparation is specified in the material and methods.<br>1. For CSR assays, cells were collected and stained with IgA-PE antibody prior to flow cytometry analysis.<br>2. For p-H3, cells were collected by trypsinization, fixed and stained with p-H3 Ser10 antibody prior to staining with Alexa Fluor 647 goat anti-rabbit secondary antibody followed by flow cytometry analysis.<br>3. For DR-GFP assay, cells were co-transfected with BPF and I-SceI, collected 72 h post transfection by trypsinization and resuspended in PBS.
Instrument	1. Fortessa analyser (BD Biosciences)<br>2. LSR II Flow Cytometer (BD Biosciences)<br>3. Attune TM NxT Acoustic Focusing Cytometer (Thermo Fisher Scientific)
Software	FlowJo version 10
Cell population abundance	No cell sorting was used in this study.
Gating strategy	For CSR, cells were first gated for live cells using FSC-A/SSC-A, followed by gating for single cells (FSC-H/FSC-W). Examples of the selection for positive cells are shown in Supplementary Figure 2c and Supplementary Figure 7e. For p-H3 data, cells were first gated for live and single cells using FSC/SSC. The selection of pH3-positive cells are shown in Supplementary Figure 5c-d, which also shows the corresponding controls for samples only stained with secondary antibodies. For DR-GFP, cells were gated for single cells and gated for BFP-positive cells after which GFP-positive cells were quantified. The gating strategy for GFP-positive cells used in Figure 4e is shown in Supplementary Figure 5i.

- [x] Tick this box to confirm that a figure exemplifying the gating strategy is provided in the Supplementary Information.
